# Compulsive Internet Pornography Use and Mental Health: A Cross-Sectional Study in a Sample of University Students in the United States

**DOI:** 10.3389/fpsyg.2020.613244

**Published:** 2021-01-12

**Authors:** Christina Camilleri, Justin T. Perry, Stephen Sammut

**Affiliations:** Department of Psychology, Franciscan University of Steubenville, Steubenville, OH, United States

**Keywords:** depression, anxiety, stress, mental health, addictive behavior, compulsivity, pornography, COVID-19

## Abstract

**Background:**

The sustained rise in negative mental health reports among university students is a source of continued global concern, and investigation continues into potential contributors to this rise. This includes the increased prevalence of risky sexual behaviors. Related is the increased prevalence of pornography use. Our study sought to explore the potential relationship between compulsive use of pornography and mental health in university students.

**Methods:**

Our sample consisted of university students (*N* = 1031; 34% male, 66% female) from Franciscan University of Steubenville, Steubenville, Ohio. An anonymous survey was sent to all students at the university over the age of 18. The survey was comprised of the following: (1) demographic questions, (2) questions on pornography use and perception, (3) a modified version of the Compulsive Internet Use Scale (mCIUS) assessing various factors associated with compulsive internet pornography use, (4) questions assessing emotional and sexual states relative to pornography use (EmSS), and (5) the 21-question version of the Depression, Anxiety and Stress Scale (DASS-21).

**Results:**

Our results indicate that 56.6% of those surveyed reported lifetime pornography use, with a significantly higher proportion of males than females reporting such use. The majority of students reported accessing pornography through internet-related technologies. Additionally, 17.0, 20.4, and 13.5% of students reported severe or extremely severe levels of depression, anxiety and stress, respectively, with compulsive pornography use significantly affecting all three mental health parameters in both sexes. Exploratory Factor Analysis identified three factors suggesting emotional coping, dependence and preoccupation for the mCIUS items and three factors reflecting interoceptive, impotent, and extrinsic characteristics for the EmSS items. Regression analysis indicated that various demographics, items pertaining to reduced control and social impairment, and other variables pertaining to pornography use predicted mental health outcomes. Faith, morals and personal motivation were the primary variables reported to help reduce pornography use.

**Conclusion:**

Our analyses indicate a significant relationship between mental health and pornography use, including behaviors reflecting behavioral addictions, highlighting the necessity for a better understanding and consideration of the potential contribution of internet pornography to negative mental health among university students.

## Introduction

Mental health issues are a growing source of global concern, especially among university students, as research indicates an increasing trend in mental health issues across this population (Macaskill, 2013; Beiter et al., 2015; Bruffaerts et al., 2018; Patterson et al., 2019; Torales et al., 2019). Given that university students are particularly prone to mental health issues, research efforts continue to investigate various factors that may potentially contribute to the observed negative mental health issues (Beiter et al., 2015; Cashwell et al., 2016; Pal Singh Balhara et al., 2019). In the general population, among the reported potential correlates are finances, childhood adversity and addictive behaviors (e.g., substance, sex and internet addictions) (Weiss, 2004; Mossakowski, 2008; Opitz et al., 2009; Ljungqvist et al., 2016; Karacic and Oreskovic, 2017; Alhassan et al., 2018; Selous et al., 2019; Wang et al., 2019). These factors are observed among university students (Cranford et al., 2009; Beiter et al., 2015; Cashwell et al., 2016; Richardson et al., 2017; Karatekin, 2018; Pal Singh Balhara et al., 2019; Tangmunkongvorakul et al., 2019), in addition to other potential correlates, including academic performance, pressure to succeed and post-graduation plans (Beiter et al., 2015).

Additionally, research indicates an increase in the prevalence of risky sexual behaviors (e.g., number of sexual partners, age of initial sexual encounter, sexting, etc.) among university students (Tyden et al., 2012; Stenhammar et al., 2015; Ingram et al., 2019; Yang et al., 2019), which have also been reported to be associated with mental health issues (Meade and Sikkema, 2007; Agardh et al., 2012; Tesfaye et al., 2019). Related is an increase in the prevalence of pornography use among this population (Carroll et al., 2008; Willoughby et al., 2014), with reports of negative effects associated with its use, including associations with other high risk sexual behaviors (Weinberg et al., 2010; Morgan, 2011; Poulsen et al., 2013; Wright, 2013a,b; Van Ouytsel et al., 2014; Braithwaite et al., 2015). This increase could potentially be associated with the significant hormonal, physical, psychological and emotional changes taking place during the adolescent years and young adulthood (Ostovich and Sabini, 2005; Fortenberry, 2013; Kar et al., 2015; Kneeland and Dovidio, 2020).

Initially being consumed primarily through magazines, patterns of pornography use have developed so that today, the internet is the primary medium for pornography consumption (D’Orlando, 2009). This shift has made pornography more accessible than in the past due to the anonymity, accessibility, and affordability the internet provides to the consumer (Cooper et al., 2000; Fisher and Barak, 2001; Price et al., 2016). Furthermore, the development of smartphones, and their substantial prevalence among young adults (Pew Research Center, 2015), has contributed to this ease of access to pornography (Bailin et al., 2014; Vanden Abeele et al., 2014). Reports of pornography use are diverse, with numbers varying from 19.0–78.4% in females and 40.0–79.0% in males (Carroll et al., 2008; Regnerus et al., 2016; Dwulit and Rzymski, 2019).

Regarding differences between males and females, research indicates differences between the sexes in sexual behaviors and attitudes (Petersen and Hyde, 2010), with males generally being more visually driven and females tending to be more emotionally driven relative to sexual behaviors (Brody, 2003; Hamann et al., 2004; Rupp and Wallen, 2008). Supporting this is research indicating that, in females, sex addiction tends to be more “relationally motivated” (McKeague, 2014). Related are differences in the prevalence of pornography use between males and females. Despite increasing reports of pornography use among women (Wright et al., 2013), the prevalence of such use remains higher in men (Regnerus et al., 2016). Additionally, research indicates a distinction in the way males and females interact with pornography, as well as differences in their views of and experiences with pornography within the context of various situations (e.g., in relationships, etc.) (Carroll et al., 2016; Döring et al., 2017). For example, males are more likely to be exposed to pornography at an earlier age, to use pornography alone, to masturbate while viewing pornography, and to see pornography use within a committed relationship as more acceptable than women (Hald, 2006; Carroll et al., 2008; Morgan, 2011; Olmstead et al., 2013; Carroll et al., 2016). Females generally view pornography as less socially acceptable than men (Carroll et al., 2008; Carroll and Lynch, 2016); however, they are more likely to consume pornography in the context of a “social” setting, such as with a romantic partner or through mediums such as sexual chat rooms (Green et al., 2012). Research indicates that women are also more likely to consume pornography in order to please their partner by consuming it together (Solano et al., 2020). Additionally, while videos remain the primary mode of consumption in both males and females, women report higher levels of consuming written pornography than men (Solano et al., 2020).

The consequences of pornography use continue to be a source of controversy as literature indicates reports of both positive (Carroll et al., 2008; Weinberg et al., 2010; Short et al., 2012; Olmstead et al., 2013; Minarcik et al., 2016) and negative (Vega and Malamuth, 2007; Padilla-Walker et al., 2010; Short et al., 2012) effects of pornography use on the consumer. Some reports indicate that, among individuals who view pornography use as acceptable, such use opens the door to sexual empowerment and autonomy (Weinberg et al., 2010; Olmstead et al., 2013). However, as previously mentioned, literature reports negative effects including increased participation in risky sexual behaviors, such as an increased number of sexual partners, sexual permissiveness, engaging in extramarital sex, and in paying for sex (Maddox et al., 2011; Gwinn et al., 2013; Poulsen et al., 2013; Wright, 2013a,b; Maas and Dewey, 2018). Additionally, although pornography depicts acts that are relational in nature, research also suggests that viewing pornography has a negative effect on relationship satisfaction, sexual satisfaction, and intimacy within heterosexual relationships, both dating and marital, particularly when the man is the pornography user (Maddox et al., 2011; Morgan, 2011; Poulsen et al., 2013; Resch and Alderson, 2013; Minarcik et al., 2016; Perry and Hayward, 2017). Moreover, pornography use has not only been associated with negative sexual behaviors, but also with binge drinking behaviors and drug use (Carroll et al., 2008; Padilla-Walker et al., 2010; Harper and Hodgins, 2016).

Specifically among younger populations, research has indicated a relationship between pornography use and both decreased friendship quality and higher levels of body monitoring in young women (Padilla-Walker et al., 2010; Maas and Dewey, 2018). Additionally, pornography use has been associated with decreased relationship quality with parents and more negative perceptions of social acceptance in both young men and women (Padilla-Walker et al., 2010). Moreover, previous literature has indicated a potential link between mental well-being and pornography use, including relative to *perceived* addiction to pornography (Grubbs et al., 2015b,c; Dalby et al., 2018).

However, while previous research has sought to investigate the relationship between pornography use, mental health and *perceived* addiction to pornography as indicated above, research specifically addressing the role of various behaviors associated with *compulsivity* rather than personal perception on the relationship between pornography use and mental health is lacking. Additionally, given the increasing reports of mental health concerns among university students, as well as the prevalence of pornography use reported among young adults and its potential to influence mental well-being, the goal of our study was to directly explore the potential relationship between behaviors reflecting compulsive use of pornography and mental health, specifically in university students. Additionally, given the consistent differences between males and females relative to sexual behaviors, including pornography use, our study also sought to investigate whether such differences persisted in the putative relationship between pornography use, compulsive behavior and mental health in university students, especially given the significant changes in method and ease of access of pornography that have occurred over the years and the uniqueness in response to stimuli, even at the neurobiological level, between the sexes.

## Materials and Methods

In compliance with Federal Law indicating that all researchers conducting testing on human participants must complete training on the protection of research subjects, all survey administrators completed the Protecting Human Research Participants Training Module provided by the NIH Office of Extramural Research. Certification is kept on file for documentation purposes. Prior to administration of the survey, Franciscan University of Steubenville Institutional Review Board (IRB) approval was obtained (#2019-07). Our study consisted of a convenience sample of university/college (undergraduate and graduate) students from Franciscan University of Steubenville, a small private Catholic university located in Steubenville, OH, United States. An anonymous survey was sent via the university student email address, to all students taking classes at Franciscan University, who were over the age of 18. Over the course of 2 weeks (October 15th – October 28th, 2019), the survey was administered through the online survey engine SurveyMonkey^®^. Prior to completing the survey, participants were directed to a consent form, which detailed the confidentiality and the nature of the study and results, and explained that participation in the study implied consent to analyze and publish the overall results. Participants who did not provide consent were directed to the *Disqualification Page*. The projected time of administration and completion of the survey was approximately 10 min. The instructions indicated to students that they should give their honest response and not spend too much time on any question. The final page of the survey also included various resources for the participants if they desired to seek assistance in regards to their pornography use.

### Exclusion Criteria

Exclusion criteria included any individual: (1) who was younger than 18 years of age (*n* = 2), (2) was not a student at Franciscan University of Steubenville (*n* = 4), (3) responded “No” (*n* = 15) or did not complete the question regarding consent (*n* = 73), (4) who did not complete the survey question regarding their age (*n* = 23), and (5) who did not provide a response for the last time they viewed pornography (*n* = 24). The final number of participants whose responses met inclusion criteria was 1031 (out of the original 1172 total respondents, i.e., 88%).

### Survey Structure

#### Demographic Questions

Demographic questions included: age, sex, class, number of semesters completed at Franciscan University, major, housing during the school year at the time of survey, and relationship status. Participants were also asked to indicate whether they were an online-only and/or a transfer student and whether they shared a room with someone during the school year.

#### Questions on Pornography Use and Perception

Participants were asked to indicate the last time they viewed internet pornography, their frequency of use during their period of most frequent use, the time of day during which they most often viewed pornography and what form of pornography they most often accessed/viewed. Questions were also asked pertaining to how and what form of pornography they were first exposed to, as well as their age of first exposure. Moreover, participants were asked to select all of the aspects that have helped them decrease their pornography use. Only participants indicating some level of lifetime use of pornography were directed to the sections of the survey associated with personal pornography use.

Additionally, the survey inquired about what percentage of both males and females at Franciscan University they thought struggled with pornography. The participants were also asked to rate, on a four-point scale (from *Not at all pornographic* to *Extremely pornographic*), how pornographic they considered various materials (e.g., Nude pictures, Cinematic sex scenes, Nude art, etc.) to be.

#### Modified Compulsive Internet Use Scale (mCIUS)

The survey also included the 13 questions of the mCIUS (Downing et al., 2014) in order to assess various factors associated with compulsive use of internet pornography. Participants were instructed to answer the questions based upon their period of most frequent use of pornography. Each mCIUS question was rated on a five-point Likert scale (from *Never* to *Very often*). In this scale, greater average scores indicate a higher compulsive use of internet pornography (Downing et al., 2014).

#### Emotional and Sexual States Questionnaire (EmSS)

Questions pertaining to emotional and sexual states (Downing et al., 2014) were also asked in order to assess when individuals were more likely to view internet pornography (e.g., with a sexual partner, bored, etc.). Two modifications were made to the original questions, the first being that *alone* was split into two separate questions: *by myself* and *lonely*, given the distinction between the two states (Algren et al., 2020). The word *Horny* was also modified to *feeling sexually aroused*. Additionally, while Downing et al. (2014) used a four-point Likert scale, our survey utilized a five-point Likert scale (*Strongly Disagree*, *Disagree*, *Neither Agree or Disagree*, *Agree*, and *Strongly Agree*) in order to provide the possibility of an individual answering neither agree nor disagree.

#### Depression, Anxiety, and Stress Scale (DASS-21)

The 21-question version of the DASS (Lovibond and Lovibond, 2004) was also included in the survey, which measures various core symptoms associated with depression (D), anxiety (A), and stress (S). Subjects were instructed to indicate how much each statement applied to them over the past week on a four-point Likert scale (from 0 = *Did not apply to me at all* to 3 = *Applied to me very much, or most of the time*). The DASS-21 is not intended to diagnose disorders related to depression, anxiety or stress. The participants’ total scores in the three criteria (D, A, and S) were categorized by severity as either “normal,” “mild,” “moderate,” “severe,” or “extremely severe,” as previously defined (Lovibond and Lovibond, 1995).

### Statistical Analysis

Analyses were conducted on all data (*n* = 1031). Using R version 3.6.2, Chi squared or Fisher’s exact test were conducted, as appropriate, to analyze differences in proportions across various factors associated with pornography use, as well as mental health parameters, across and between the sexes. Additionally, independent measures *t*-tests and two-way independent measures ANOVAs were conducted, using SigmaPlot version 14.0, to analyze differences in mental health parameters based on pornography use, across the sexes. Tukey *post hoc* analysis was conducted where appropriate. Exploratory factor analysis (EFA) was utilized in our study as we sought to explore the relationship between various variables, and uncover specific potential factors pertaining to compulsive behavior (mCIUS), emotional and sexual states (EmSS) and pornography use, rather than attempting to confirm a specific hypothesis in relation to the various factors and pornography use. Using Jamovi 1.1.7, Bartlett’s test of sphericity and the Kaiser–Meyer–Olkin measure of sampling adequacy were utilized to determine the factorability of the data from both the mCIUS and EmSS items. Based on the results from the previous two tests, EFA, also conducted using Jamovi 1.1.7, was utilized to analyze response patterns within the mCIUS items and EmSS items, separately. Backward stepwise elimination regression was utilized to determine the relationship between various demographics, various aspects of pornography use, and mental health parameters (D, A, S). Two separate models were utilized: model 1 included the mCIUS items as predictor variables, while model 2 addressed the EmSS items as predictor variables. For both models, various demographics measured, as well as aspects related to pornography use, were also included as additional predictor variables, and the depression, anxiety and stress scores were considered dependent variables.

## Results

### Demographics

Consistent with national trends (e.g., Fry, 2019), the distribution of the participants in the survey was 34% male and 66% female, which resembled the sex distribution of the student body at Franciscan University. The data for the demographic questions pertaining to age, class, number of semesters completed (Semesters completed), living status and relationship status are included in Table 1 shown across sex. Students were additionally asked to indicate whether or not they were an online-only student (Online-only), a transfer student (Transfer), and whether or not they shared a room with someone during the school year (Share room). This data is also included in Table 1.

**TABLE 1:** Summary of demographic variables.

**Variable**	**Male (M) (*n* = 347; 34%) *n* (%)**	**Female (F) (*n* = 684; 66%) *n* (%)**
**Age** 18 19 20 21 22 23 24+	57 (16.4) 63 (18.2) 50 (14.4) 49 (14.1) 24 (6.9) 13 (3.7) 91 (26.2)	120 (17.5) 137 (20.0) 114 (16.7) 103 (15.1) 44 (6.4) 15 (2.2) 151 (22.1)
**Class** Freshman Sophomore Junior Senior Graduate	82 (23.6) 61 (17.6) 46 (13.3) 66 (19.0) 92 (26.5)	152 (22.2) 144 (21.1) 117 (17.1) 123 (18.0) 148 (21.6)
**Online-only** Yes No	81 (23.3) 266 (76.7)	134 (19.6) 550 (80.4)
**Transfer** Yes No	44 (12.7) 303 (87.3)	109 (15.9) 575 (84.1)
**Semesters completed** <1 1–2 3–4 5–6 7–8 9+	110 (31.7) 89 (25.6) 72 (20.7) 47 (13.5) 16 (4.6) 13 (3.7)	215 (31.4) 188 (27.5) 130 (19.0) 101 (14.8) 26 (3.8) 24 (3.5)
**Living status** Main Campus – M/F only Dorms Main Campus – Co-ed Dorms Main Campus – Assisi Heights Lower Campus Off Campus Gaming, Austria	116 (33.4) 38 (11.0) 26 (7.5) 16 (4.6) 145 (41.8) 6 (1.7)	235 (34.4) 103 (15.1) 73 (10.7) 9 (1.3) 242 (35.4) 22 (3.2)
**Share room** Yes No	230 (66.3) 117 (33.7)	475 (69.4) 209 (30.6)
**Relationship status** Single In a relationship Married Discerning religious life/Priesthood Priest or other religious Divorced/Separated Widow(er)	175 (50.4) 81 (23.3) 63 (18.2) 22 (6.3) 4 (1.2) 2 (0.6) 0 (0.0)	433 (63.3) 123 (18.0) 84 (12.3) 22 (3.2) 10 (1.5) 9 (1.3) 3 (0.4)

### Current Pornography Use

Given that the proportion of participants reporting lifetime pornography use was not significantly different relative to those reporting having never used pornography [χ^2^(1, *N* = 1031) = 0.0, *p* > 0.05 and χ^2^(1, *N* = 1031) = 0.7, *p* > 0.05, respectively] in online-only versus residential (i.e., not online-only) students, as well as those who transferred into the university relative to those who did not, the data analysis presented below does not make a distinction based on these two variables.

For questions containing an “*Other (please specify)*” answer choice, due to the small number of participants selecting this option, and the variety and ambiguity of the responses given, which could potentially confound interpretation, these responses were excluded from analyses and percentages shown.

#### Last Reported Pornography Use

From the 1031 respondents, a significantly higher percentage [χ^2^(1, *N* = 1031) = 35.9, *p* < 0.001] indicated lifetime use of pornography (56.6%) relative to those who reported never using pornography (43.4%).

**FIGURE 1:**
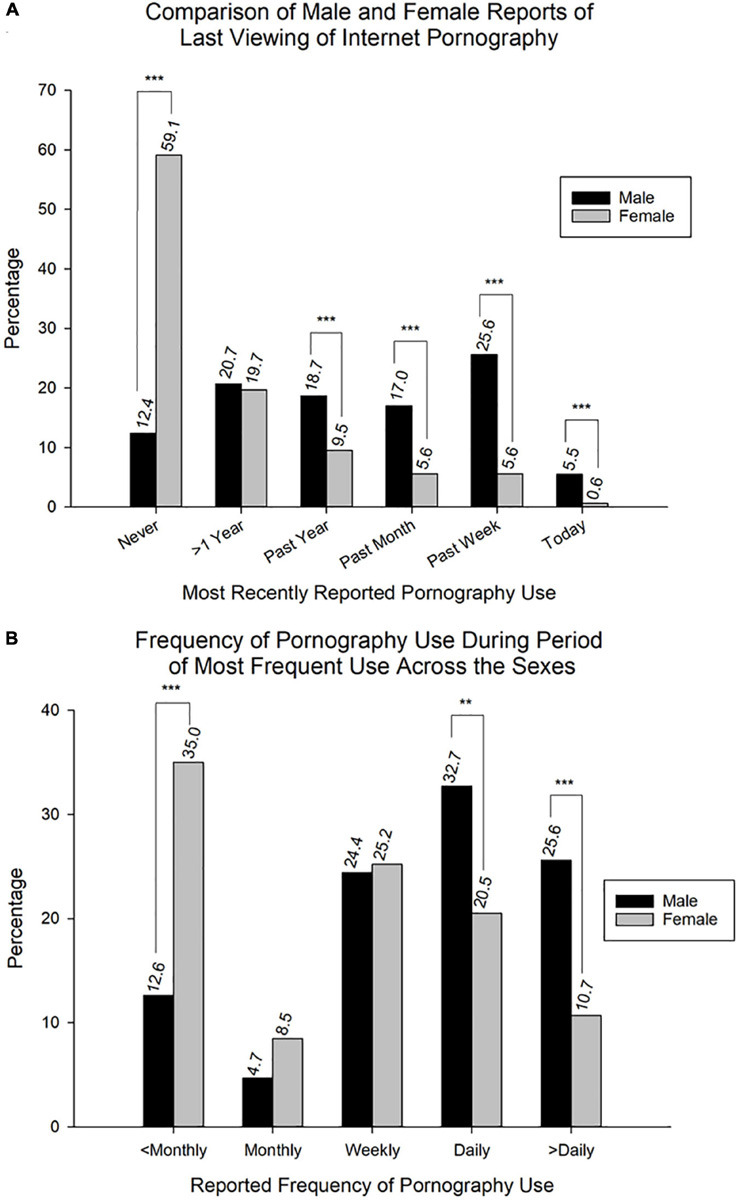
Reported pornography use across the sexes. **(A)** Comparison of last viewing of internet pornography reported across the sexes (*N* = 1031; Male: *n* = 347; Female: *n* = 684). Abbreviations for the last reported pornography use: *Never*, never having viewed pornography; >*1 Year*, more than a year ago; *Past Year*, within the past year; *Past Month*, within the past month; *Past Week*, within the past week; *Today*, today. **(B)** Reported frequency of pornography use during period of most frequent use in both males and females (*N* = 488; Male: *n* = 254; Female: *n* = 234). Abbreviations for reported frequency of pornography use: <*Monthly*, less than monthly; *Monthly*, monthly; *Weekly*, weekly; *Daily*, daily; >*Daily*, multiple times daily. Data is expressed as percentage of participants responding to specific options. **p < 0.01, ***p < 0.001.

Also consistent with current trends (Carroll et al., 2008; Regnerus et al., 2016; Dwulit and Rzymski, 2019), the proportion of males (87.6%) reporting having used pornography was significantly higher [χ^2^(1, *N* = 1031) = 202.3, *p* < 0.001] than that of females (40.9%). The distribution of respondents who reported last viewing pornography was distributed as follows: *I’ve never viewed pornography* (*Never*, 43.4%), *More than a year ago* (>*1 Year*, 20.1%), *Within the past year* (*Past Year*, 12.6%), *Within the past month* (*Past Month*, 9.4%), *Within the past week* (*Past Week*, 12.3%), and *Today* (2.2%). A more detailed breakdown of pornography use across the sexes is provided in Figure 1A [χ^2^(1, *N* = 1031) = 202.3 (*Never*), 0.1 (>*1 Year*), 17.0 (*Past Year*), 34.1 (*Past Month*), 84.2 (*Past Week*), 23.1 (*Today*)].

Within *Males*, relative to those who reported having never viewed pornography (12.4%), a significantly higher proportion reported their most recent pornography use as being *More than a year ago* (20.7%, *p* < 0.05) and *Within the past week* (25.6%, *p* < 0.001), while a significantly lower percentage reported having viewed pornography *Today* (5.5%, *p* < 0.05). The percentage of those reporting use in the past year or past month was not significantly different (*p* > 0.05) from those reporting having never viewed pornography [χ^2^(5, *N* = 347) = 61.3, *p* < 0.001].

In contrast, a significantly higher proportion [χ^2^(5, *N* = 684) = 1164.1, *p* < 0.001] of females reported never having viewed pornography relative to all other options for having viewed pornography (all *p* < 0.001).

#### Frequency of Pornography Use

Of the 584 respondents confirming having previously used pornography, 488 completed the question pertaining to frequency of use during the period of most frequent use. The frequency of use reported was distributed as follows: *Less than monthly* (<*Monthly*, 23.4%), *Monthly* (6.6%), *Weekly* (24.8%), *Daily* (26.8%), and *Multiple times daily* (>*Daily*, 18.4%). Further details pertaining to frequency of use across the sexes are shown in Figure 1B [χ^2^(1, *N* = 488) = 33.0 (<*Monthly*), 2.3 (*Monthly*), 0.0 (*Weekly*), 8.6 (*Daily*), 17.0 (>*Daily*)].

The proportion of males reporting *Less than monthly* (12.6%) and *Monthly* (4.7%) use was significantly lower (all *p* < 0.01) than *Weekly* (24.4%), *Daily* (32.7%), and *Multiple times daily* (25.6%). Additionally, the proportion reporting *Monthly* use was significantly lower (*p* < 0.05) than those reporting *Less than monthly* use [χ^2^(4, *N* = 254) = 79.3, *p* < 0.001].

Relative to females, the proportion of those reporting *Less than monthly* (35.0%) use was significantly higher than *Monthly* (8.5%, *p* < 0.001), *Daily* (20.5%, *p* < 0.01), and *Multiple times daily* (10.7%, *p* < 0.001), while the proportion of those reporting *Weekly* (25.2%) use showed a tendency toward significance (*p* = 0.08). *Monthly* use was significantly lower than both *Weekly* (*p* < 0.001) and *Daily* (*p* < 0.01), but was not significantly different from *Multiple times daily* (*p* > 0.05). Additionally, *Weekly* and *Daily* use were both significantly higher than *Multiple times daily* (*p* < 0.001 and *p* < 0.05, respectively). However, *Weekly* use was not significantly different from *Daily* use (*p* > 0.05) [χ^2^(4, *N* = 234) = 69.0, *p* < 0.001].

#### Time of Day of Most Frequent Pornography Use

The question inquiring about the time of day that pornography was most often viewed was answered by 488 respondents. Given that there was no significant difference between the male and female responses [χ^2^(1, *N* = 488) = 2.3, 0.1, and 1.0 for *Before the start of your day*, *During your day* and *End of your day*, respectively, all *p* > 0.05], the combined data for the sexes was analyzed. The largest proportion of respondents reported most often viewing pornography at the end of their day (71.1%), which was significantly higher than both viewing during their day (24.2%, *p* < 0.001) and before the beginning of their day (4.7%, *p* < 0.001). The percentage of those reporting viewing pornography during their day was also significantly higher than those reporting viewing before the beginning of their day (*p* < 0.001).

#### How Pornography Was Accessed

**FIGURE 2:**
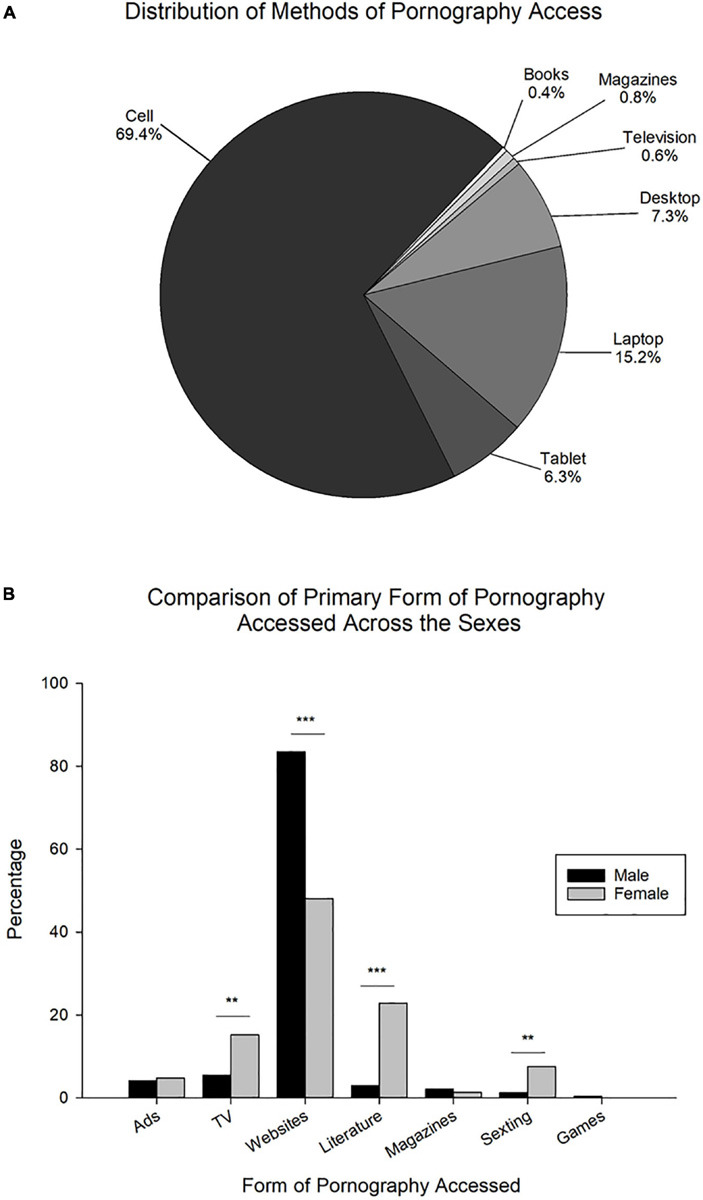
Details of pornography use during period of most frequent use. **(A)** Distribution of methods of pornography use. Given that no significant differences were observed between the sexes, data is shown as a percentage of males and females combined indicating specific method of access. *Cell*, cell phone; *Books*, physical (paper) books; *Magazines*, magazines; *Television*, television; *Desktop*, desktop computer; *Laptop*, laptop computer; *Tablet*, tablets (e.g., Kindle, iPad, etc.) (*N* = 488). **(B)** Comparison of the primary form of pornography accessed during period of most frequent use across the sexes (*N* = 488; Male: *n* = 254; Female: *n* = 234). *Ads*, advertisements on the internet; *TV*, TV/Movies; *Websites*, adult websites (e.g., pornography websites); *Literature*, Adult/Erotic literature; *Magazines*, pornographic magazines, *Sexting*, sexting/phone sex/hotlines/Snapchat, *Games*, adult video games. Data is expressed as percentage of participants responding to a specific answer choice. **p < 0.01, ***p < 0.001.

Pertaining to how pornography was most often accessed, there was no significant difference between the responses of males and females (χ^2^ or Fisher’s Test, all *p* > 0.05). Thus, the combined data for males and females was analyzed (Figure 2A). The primary methods of access for pornography reported to be utilized by a majority of respondents were internet-related technologies (Cell phone, laptop and desktop computers, and tablets; 98.2%). Specifically, access through the *Cell phone* (69.4%) was significantly higher than all other options (all *p* < 0.001). The next highest method of access reported was *Laptop computer* (15.2%), which was significantly higher than *Tablets (e.g., Kindle, iPad, etc.)* (6.3%), *Desktop computer* (7.3%), *Television* (0.6%), *Magazines* (0.8%), and *Physical (paper) books* (0.4%), all *p* < 0.001. Additionally, those reporting the use of both tablets and desktop computer were significantly higher than *Television*, *Magazines*, and *Physical (paper) books* (all *p* < 0.001). All other comparisons were not significant (all *p* > 0.05). In the context of this question, 8 participants responded *Other*, making up 1.6% of total respondents to this question.

#### Form of Pornography Accessed

In relation to the form of pornography that was predominantly accessed by the individual sexes, significant differences were present between the sexes {Figure 2B, χ^2^(1, *N* = 447) = 0.0 (*Advertisements on the Internet*), 10.6 (*TV/Movies*), 61.6 [*Adult websites (e.g., pornography websites)*], 39.1 (*Adult/Erotic literature*), 9.5 (*Sexting/phone sex/hotlines/Snapchat*)}.

Pertaining to males, a majority of respondents (83.5%) indicated that they most often accessed *Adult websites (e.g., pornography websites)*, which was significantly higher than all other options [*Advertisements on the Internet* (4.2%), *TV/Movies* (5.5%), *Adult/Erotic literature* (3.0%), *Pornographic Magazines* (2.1%), *Sexting/phone sex/hotlines/Snapchat* (1.3%), *Adult video games* (0.4%); all *p* < 0.001]. The percentage of those reporting access through *TV/Movies* was significantly higher than both sexting, etc. (*p* < 0.05) and adult video games (*p* < 0.01). Additionally, access through *Advertisements on the Internet* was significantly higher than *Adult video games* (*p* < 0.05). All other comparisons were not significant (*p* > 0.05).

Like males, a majority of females (48.1%) reported most often accessing pornography through adult websites. This was significantly higher than all other options [*Advertisements on the Internet* (4.8%), *TV/Movies* (15.2%), *Adult/Erotic literature* (22.9%), *Pornographic Magazines* (1.4%), *Sexting/phone sex/hotlines/Snapchat* (7.6%), *Adult video games* (0.0%); all *p* < 0.001]. This was followed by adult literature, which was significantly higher than internet advertisements, pornographic magazines, sexting, etc. and adult video games (all *p* < 0.001). The proportion of females reporting access through TV/Movies, the third highest form most often accessed, was significantly higher than advertisements on the internet, pornographic magazines, and adult video games (*p* < 0.001), as well as sexting, etc. (*p* < 0.05). Pornography access through *Sexting/phone sex/hotlines/Snapchat* was significantly higher than both pornographic magazines (*p* < 0.01) and adult video games (*p* < 0.001). Finally, access through internet advertisements was significantly higher than adult video games (*p* < 0.01). All other comparisons were not significant (all *p* > 0.05).

Pertaining to the question inquiring into the form of pornography most often accessed, 41 participants responded *Other (please specify)*, making up 8.4% of the total respondents to this question.

### First Exposure to Pornography

As above, for questions containing an “*Other (please specify)*” answer choice, these responses were excluded from analyses and percentages shown.

#### Age of First Exposure to Pornography

**FIGURE 3:**
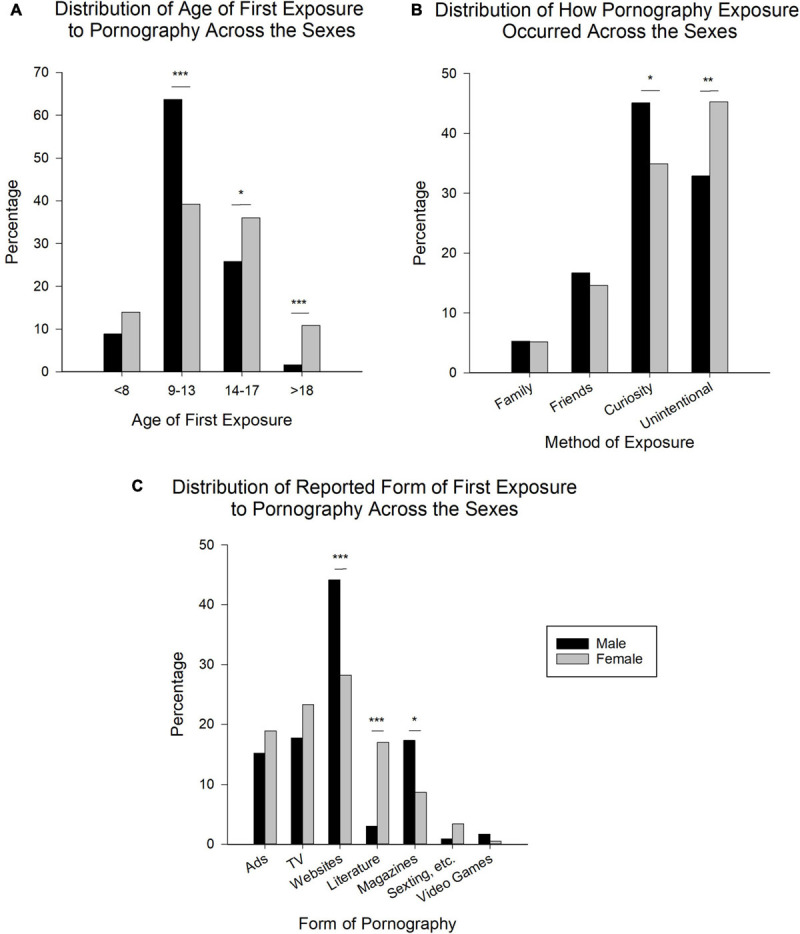
First exposure to pornography across the sexes. **(A)** Age of first exposure to pornography across the sexes. <*8*, 8 years or younger; *9–13*, 9–13 years of age; *14–17*, 14–17 years of age; >*18*, 18 years or older. **(B)** Distribution of how exposure to pornography occurred in males and females. *Family*, through family; *Friends*, through friends; *Curiosity*, personal curiosity; *Unintentional*, unintentional exposure. **(C)** Form of pornography to which first exposure occurred across the sexes. *Ads*, pop-ups/advertisements on the Internet; *TV*, television/movies; *Websites*, adult websites (e.g., pornography websites); *Literature*, adult/erotic literature; *Magazines*, pornography magazines; *Sexting, etc.*, sexting/phone sex/hotlines/Snapchat; *Video Games*, adult video games. Data is expressed as percentage of respondents indicating specific answer choices (*N* = 470; Male: *n* = 248; Female: *n* = 222). *p < 0.05, **p < 0.01, ***p < 0.001.

Analysis revealed significant differences between males and females relative to the reported age of first exposure to pornography [Figure 3A, χ^2^(1, *N* = 470) = 2.5 (*8 or younger*), 27.3 (*9–13*), 5.3 (*14–17*), 16.1 (*18 or older*)].

A majority of males (63.7%) reported *9–13* as the age of first exposure to pornography, which was significantly higher than all other options [*8 or younger* (8.9%), *14–17* (25.8%), *18 or older* (1.6%), all *p* < 0.001]. All other comparisons of age of first exposure for males were significant (all *p* < 0.001) [χ^2^(3, *N* = 248) = 305.0, *p* < 0.001].

Relative to females, similar to males, the mode age of first exposure was also *9–13* (39.2%). While this was not significantly higher than those reporting *14–17* (36.0%, *p* > 0.05), it was significantly higher than both *8 or younger* (14.0%) and *18 or older* (10.8%), both *p* < 0.001. Additionally, the proportion of females reporting *14–17* was also significantly higher than both *8 or younger* and *18 or older*, both *p* < 0.001. There was no significant difference (*p* > 0.05) between females who reported *8 or younger* relative to *18 or older* as the age in which they were first exposed to pornography [χ^2^(3, *N* = 222) = 76.5, *p* < 0.001].

#### How Pornography Exposure Occurred

Regarding how the first exposure to pornography took place, significant differences were present between the sexes [Figure 3B, χ^2^(1, *N* = 458) = 0.0 (*Through family*), 0.2 (*Through Friends*), 4.5 (*Personal Curiosity*), 6.8 (*Unintentional Exposure*)].

In both males and females, *Personal curiosity* (Male: 45.1%; Female: 34.9%) and *Unintentional exposure* (Male: 32.9%; Female: 45.3%) were the primary methods through which first exposure occurred. However, in males, personal curiosity was significantly higher than unintentional exposure (*p* < 0.01), while in females, unintentional exposure was significantly higher than personal curiosity (*p* < 0.05). Both of these methods of exposure were significantly higher, in both sexes, than *Through family* (Male: 5.3%; Females: 5.2%) and *Through friends* (Male: 16.7%; Female: 14.6%), all *p* < 0.001. Additionally, in both sexes, exposure through friends was significantly higher than exposure through family (Males: *p* < 0.001; Females: *p* < 0.01). Pertaining to this question, 12 participants (2.6% of the total respondents to this question) selected *Other* [Male: χ^2^(3, *N* = 246) = 121.5, Female: χ^2^(3, *N* = 212) = 114.2, both *p* < 0.001].

#### First Exposure: Form of Pornography

Relative to the form of pornography to which the respondents were first exposed, analysis revealed significant differences between males and females within the various forms of exposure [Figure 3C, χ^2^(1, *N* = 437) = 0.9 (*Pop-ups/Advertisements on the Internet*), 1.7 (*TV/Movies*), 11.3 (*Adult websites (e.g., pornography websites*), 22.8 (*Adult/Erotic literature*), 6.2 (*Pornographic Magazines*)].

Pertaining to males, 44.2% reported *Adult websites (e.g., pornography websites)* as the form of pornography to which they were first exposed. This was significantly higher than all other forms: *Pop-ups/advertisements on the Internet*, 15.2%; *Television/Movies*, 17.7%; *Adult/erotic literature*, 3.0%; *Pornographic magazines*, 17.3%; *Sexting/phone sex/hotlines/Snapchat*, 0.9% and *Adult video games*, 1.7%, all *p* < 0.001. The percentage of males reporting *Television/Movies*, *Pornographic magazines* and *Pop-ups/advertisements on the Internet* was significantly higher (all *p* < 0.001) than adult literature, sexting, etc., and adult video games. All other comparisons were not significant (*p* > 0.05).

Similar to males, adult websites was the highest reported form of pornography to which females were first exposed (28.2%), which was significantly higher than all other forms [Pop-ups, etc. (18.9%) and adult literature (17.0%), both *p* < 0.05; Magazines (8.7%), sexting, etc. (3.4%) and adult video games (0.5%), all *p* < 0.001], except TV/movies (23.3%, *p* > 0.05). Pop-ups, etc., TV/movies and adult literature were all significantly higher than sexting, etc. and adult video games, all *p* < 0.001, as well as pornographic magazines (relative to Pop-ups, etc. *p* < 0.01, TV/movies, *p* < 0.001 and adult literature, *p* < 0.05). Additionally, the proportion of those reporting pornographic magazines was significantly higher than both sexting, etc., *p* < 0.05, and adult video game, *p* < 0.001. All other comparisons were not significant (*p* > 0.05). Of the total respondents, 33 (7.0%) selected *Other (please specify)* in regards to the form of pornography to which they were first exposed.

### mCIUS Questionnaire

**FIGURE 4:**
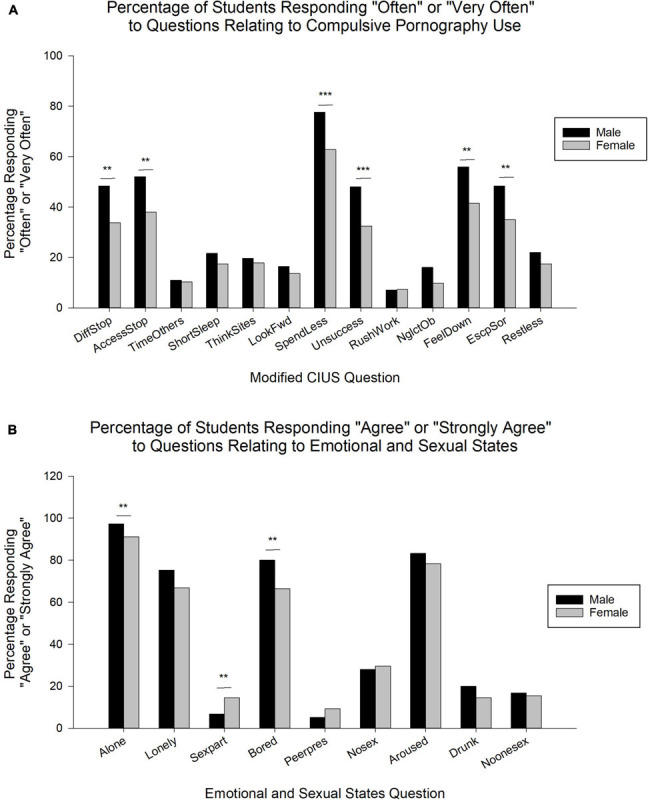
**(A)** Percentage of males and females responding “Often” or “Very Often” to items of the modified Compulsive Internet Use Scale related to pornography use. *DiffStop*, difficulty to stop accessing pornography websites; *AccessStop*, access despite intention to stop; *TimeOthers*, access pornography over spending time with others; *ShortSleep*, short of sleep due to pornography use; *ThinkSites*, think about websites when not online; *LookFwd*, look forward to next session of use; *SpendLess*, think it is necessary to spend less time; *Unsuccess*, unsuccessful at spending less time; *RushWork*, rush work to view pornography; *NglctOb*, neglect obligations due to pornography; *FeelDown*, use pornography when feeling down; *EscpSor*, use pornography to escape negative feelings; *Restless*, restless/frustrated/irritated when unable to view pornography. Data is expressed as percentage of respondents indicating “Often” or “Very Often” (*N* = 488; Male: *n* = 254; Female: *n* = 234). **(B)** Distribution of participants responding “Agree” or “Strongly Agree” to the items related to emotional and sexual states relative to pornography use across the sexes, indicating that they were more likely to view Internet pornography when by themselves (*Alone*), feeling lonely (*Lonely*), with a sexual partner (*Sexpart*), feeling bored (*Bored*), peer pressured (*Peerpres*), not having had sex in a while (*Nosex*), feeling sexually aroused (*Aroused*), drunk or under effects of drugs (*Drunk*), and unable to find someone to have sex with (*Noonesex*). Data is expressed as percentage of respondents indicating “Agree” or “Strongly Agree” (*N* = 476; Male: *n* = 250; Female: *n* = 226). **p < 0.01, ***p < 0.001.

In general, the trend of the proportion of participants responding “*Often*” or “*Very Often*” for the questions relating to compulsive pornography use was similar for both sexes. Participants in both sexes most prominently selected “*Often*” or “*Very Often*” for the questions indicating that they: (1) thought they should spend less time on pornography websites (SpendLess, Sexes combined: 70.5%; Male: 77.6%, Female: 62.8%), (2) accessed the websites when feeling down (FeelDown, Sexes combined: 49.0%; Male: 55.9%, Female: 41.5%), (3) continued to access the websites despite their intention to stop (AccessStop, Sexes combined: 45.3%; Male: 52.0%, Female: 38.0%), (4) accessed the websites to escape/get relief from negative feelings (EscpSor, Sexes combined: 42.0%; Male: 48.4%, Female: 35.0%), (5) found it difficult to stop accessing the websites when online (DiffStop, Sexes combined: 41.4%; Male: 48.4%, Female: 33.8%), and (6) unsuccessfully tried to spend less time on the websites (Unsuccess, Sexes combined: 40.6%; Male: 48.0%, Female: 32.5%). Statistically significant differences were also present between the greater proportion of males than females reporting “*Often*” or “*Very Often*” for these specific items in the mCIUS [χ^2^(1, *N* = 488) = 10.2 (DiffStop), 9.0 (AccessStop), 9.6 (FeelDown), 8.4 (EscpSor), *p* < 0.01; 12.0 (Spendless), 11.6 (Unsuccess), *p* < 0.001]. All other comparisons were not significant (*p* > 0.05). These differences and the results of the remaining questions not addressed above are described in Figure 4A and Supplementary Table 1.

Based on previous literature pertaining to internet use (Guertler et al., 2014; Yong et al., 2017; Fuchs et al., 2018) and taking into consideration the fact that the mCIUS consists of 13 questions (Downing et al., 2014), in contrast to the original 14-item survey (Meerkerk et al., 2009), categorization of severity was set at a cut-off point of 26 points (greater than or equal to 26; based on the response of at least *sometimes* for every item of the mCIUS) identifying *addictive pornography use*, 20–25 as *problematic pornography use*, and <20 as normal. Under this categorization, 57.0% of the respondents to the mCIUS displayed problematic and addictive pornography use (16.6 and 40.4%, respectively).

#### Exploratory Factor Analysis for the mCIUS

An exploratory factor analysis (EFA) using a principal-axis factor extraction (Costello and Osborne, 2005; Baglin, 2014) was utilized to investigate the factor structure of the mCIUS survey items. Parallel analysis (Costello and Osborne, 2005; Baglin, 2014) recommended a three-factor solution (Table 2). Given the high correlation of the items, a ‘promax’ (oblique) rotation (Costello and Osborne, 2005; Baglin, 2014) was utilized for interpretation of the three factors. This rotation had sums of squared loadings ranging from 1.81 to 4.16. The correlation coefficients between factors ranged from 0.699 – 0.755.

**TABLE 2:** Summary of Exploratory Factor Analysis results pertaining to items of the modified Compulsive Internet Use Scale using the principal axis factoring extraction method in combination with a promax rotation (*n* = 488).

**Factor Loadings**				
	**Factor**			
	**Preoccupation**	**Dependence**	**Emotional coping**	**Uniqueness**

**DiffStop**		0.522		0.4728
**AccessStop**		0.730		0.2993
**TimeOthers**	0.865			0.2996
**ShortSleep**	0.637			0.4824
**ThinkSites**	0.662			0.4390
**LookFwd**	0.776			0.4652
**SpendLess**		0.732		0.5598
**Unsuccess**		0.880		0.2927
**RushWork**	0.877			0.3778
**NglctOb**	0.795			0.3844
**FeelDown**			0.878	0.1453
**EscpSor**			0.976	0.0968
**Restless**	0.584			0.4302

**DiffStop*, difficulty to stop accessing pornography websites; *AccessStop*, access despite intention to stop; *TimeOthers*, access pornography over spending time with others; *ShortSleep*, short of sleep due to pornography use; *ThinkSites*, think about websites when not online; *LookFwd*, look forward to next session of use; *SpendLess*, think it is necessary to spend less time; *Unsuccess*, unsuccessful at spending less time; *RushWork*, rush work to view pornography; *NglctOb*, neglect obligations due to pornography; *FeelDown*, use pornography when feeling down; *EscpSor*, use pornography to escape negative feelings; *Restless*, restless/frustrated/irritated when unable to view pornography.*

The first factor, identified as “Preoccupation,” included preferring to access the websites instead of spending time with others (TimeOthers), being short of sleep due to being up viewing the websites (ShortSleep), thinking about the websites even when not online (ThinkSites), looking forward to the next internet session accessing the websites (LookFwd), rushing work in order to access the websites (RushWork), preferring to access the websites while neglecting daily obligations (NglctOb), and feeling restless, frustrated or irritated when unable to access the websites (Restless). The second factor was identified as “Dependence,” and included DiffStop, AccessStop, SpendLess, and Unsuccess. Finally, the third factor, identified as “Emotional Coping,” consisted of FeelDown and EscpSor. The identification of the factors will be further addressed in the Discussion.

### EmSS Questionnaire

Overall, the general trend of the proportion of participants responding “*Agree*” or “*Strongly Agree*” for the questions regarding emotional and sexual states relative to pornography use was similar between males and females. Participants in both sexes most predominately reported “*Agree*” or “*Strongly Agree*” for the questions indicating that they were *more likely to view Internet pornography when*: (1) they were by themselves (Alone, Sexes combined: 94.3%; Male: 97.2%, Female: 91.2%), (2) they were feeling sexually aroused (Aroused, 80.9%), (3) they were bored (Bored, Sexes combined: 73.5%; Male: 80.0%, Female: 66.4%), and (4) they felt lonely (Lonely, 71.2%). There were, however, statistically significant differences between the proportion of males and females reporting “*Agree*” or “*Strongly Agree*” for specific items in the EmSS. Specifically, more males than females were likely to utilize pornography when alone [χ^2^(1, *N* = 476) = 7.0] or feeling bored [χ^2^(1, *N* = 476) = 10.6], both *p* < 0.01, while a significantly higher proportion of females than males [χ^2^(1, *N* = 476) = 6.9, *p* < 0.01] reported using pornography when with a sexual partner (Male: 6.8%, Female: 14.6%). All other comparisons were not significant (*p* > 0.05). These differences and the results of the remaining items not addressed above are described in Figure 4B and Supplementary Table 2.

#### Exploratory Factor Analysis for the EmSS

Once again, EFA using a principal-axis factor extraction was utilized to investigate the factor structure of the items pertaining to emotional and sexual states. The parallel analysis indicated the presence of three factors (Table 3). Given that various dimensions of the data were non-orthogonal, an oblique (‘promax’) rotation was utilized. This rotation had sums of squared loadings ranging from 0.923 to 1.498. The correlation coefficients between factors ranged from 0.240 to 0.679.

**TABLE 3:** Summary of Exploratory Factor Analysis results pertaining to items of the Emotional and Sexual State Scale using the principal axis factoring extraction method in combination with a promax rotation (*n* = 476).

**Factor Loadings**				
	**Factor**			
	**Interoceptive**	**Impotent**	**Extrinsic**	**Uniqueness**

**Alone**	0.667			0.542
**Lonely**	0.681			0.598
**Sexpart**			0.467	0.703
**Bored**	0.554			0.676
**Peerpres**			0.586	0.739
**Nosex**		1.043		0.182
**Aroused**	0.461			0.674
**Drunk**			0.490	0.621
**Noonesex**		0.511		0.460

*Abbreviations pertaining to using internet pornography when *Alone*, by themselves; *Lonely*, feeling lonely; *Sexpart*, with a sexual partner; *Bored*, feeling bored; *Peerpres*, peer pressured; *Nosex*, not having had sex in a while; *Aroused*, feeling sexually aroused; *Drunk*, drunk or under effects of drugs; and *Noonesex*, unable to find someone to have sex with.*

The first factor was identified as “Interoceptive,” reflecting items related to circumstances that primarily involve the individuals themselves and stemming from internal feelings. These included Alone, Lonely, Bored, and Aroused. The second factor, identified as “Impotent,” reflected the increased likelihood of pornography use associated with the absence of possibilities to engage in sexual intercourse, specifically, not having sex in a while (Nosex) and not finding someone to engage in sexual intercourse with (Noonesex). Finally, the third factor, “Extrinsic,” appeared to reflect situations that involved external influences, including being with a sexual partner (Sexpart), being peer pressured (Peerpres) and being drunk or feeling the effects of drugs/illicit substances (Drunk).

### DASS-21

Based on the scoring of the DASS-21 (Lovibond and Lovibond, 2004), from the participants who completed this section of the survey (*n* = 872), 55.4, 56.0, and 63.5% of all participants fell under the “normal” category of depression, anxiety and stress, respectively. Additionally, a considerable percentage of participants reported symptoms of “severe” or “extremely severe” levels of depression (17.0%), anxiety (20.4%), and stress (13.5%) (see Supplementary Figure 1).

Analysis revealed no significant differences (all *p* > 0.05) between males and females across the various levels (“normal,” “mild,” etc.) of depression. However, a significantly higher proportion of males reported “normal” levels of both anxiety (62.2%) and stress (69.1%) relative to females (A: 53.0%; S: 60.9%), χ^2^(1, *N* = 872) = 6.1 and 5.0, respectively, both *p* < 0.05. Additionally, a significantly higher proportion [χ^2^(1, *N* = 872) = 4.1, *p* < 0.05] of females (22.4%) than males (16.2%) reported either “severe” or “extremely severe” anxiety. A significantly higher percentage [χ^2^(1, *N* = 872) = 4.2, *p* < 0.05] of females (15.5%) indicated a “moderate” level of stress relative to that of males (10.1%). All other comparisons were not significantly different (all *p* > 0.05).

### Mental Health (D, A, S) and Pornography Use

#### Last Reported Pornography Use and Mental Health

Analysis was conducted to assess the influence of last reported pornography use on mental health, as measured by the DASS-21. The average D, A, S scores for students reporting pornography use were significantly higher [*t*(870) = −5.55 and −3.81 for D and A, respectively, both *p* < 0.001; *t*(870) = −3.14 for S, *p* < 0.01] than those reporting never viewing pornography.

**FIGURE 5:**
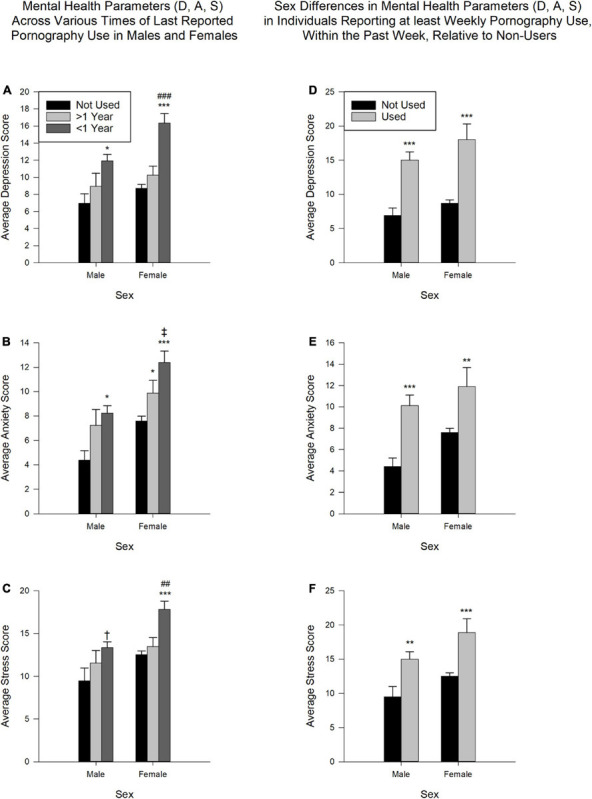
Mental health parameters in males and females relative to reported pornography use. **(A–C)** Depression **(A)**, anxiety **(B)**, and stress **(C)** scores across various times of last reported pornography use across the sexes (*N* = 872; Male: *n* = 278; Female: *n* = 594). *Not Used*, never having viewed pornography; >*1 Year*, more than a year ago; <*1 Year*, within the past year (i.e., Today, Within the past week, Within the past month, Within the past year). Relative to *Not Used*: *p < 0.05, ***p < 0.001, ^†^0.05 < *p* < 0.1. Relative to >*1 Year*: ^##^*p* < 0.01, ^###^*p* < 0.001, ^‡^0.05 < *p* < 0.1. **(D–F)** Depression **(D)**, anxiety **(E)**, and stress **(F)** scores in participants reporting at least weekly pornography use, within the past week, relative to non-users (*N* = 531; Male: *n* = 124; Female: *n* = 407). *Not Used*, never having viewed pornography; *Used*, viewed pornography in the past week, at least weekly. Data is expressed as mean ± SEM. Relative to *Not Used*: **p < 0.01, ***p < 0.001.

Furthermore, the results indicated a significant effect in all three mental health parameters (D, A, S) across sex [D: *F*(1,866) = 7.80, *p* < 0.01; A: *F*(1,866) = 18.73, *p* < 0.001; S: *F*(1,866) = 13.35, *p* < 0.001] and the last reported pornography use [D: *F*(2,866) = 22.04; A: *F*(2,866) = 11.97; S: *F*(2,866) = 12.15; all *p* < 0.001], but not in the interaction of sex and last reported use [D: *F*(2,866) = 1.48; A: *F*(2,866) = 0.39; S: *F*(2,866) = 0.88; all *p* > 0.05]. Depression, anxiety and stress scores (mean and SEM), for both males and females, across times of last reported pornography use are shown in Figures 5A–C.

Pertaining to males, significantly higher scores were observed in depression and anxiety (both *p* < 0.05) in those reporting using pornography in the previous year (*Today, Within the past week, Within the past month, Within the past year*) than those reporting having never used pornography. The same comparison, in relation to stress scores, indicated a tendency toward significance (*p* = 0.06). All other comparisons were not significant (*p* > 0.05).

In relation to females, significantly higher scores in all three mental health measures (all *p* < 0.001) were reported in those reporting pornography use in the previous year relative to those reporting never having used. The scores for depression and stress were also significantly higher (*p* < 0.001 and *p* < 0.01, respectively) in those reporting using pornography in the previous year relative to those reporting using pornography *More than a year ago*. While a similar trend was observed in the anxiety scores, statistically, this difference was a tendency toward significance (*p* = 0.08). Additionally, the anxiety scores for those reporting having used pornography *More than a year ago* was also significantly higher than those who reported having never used pornography (*p* < 0.05). All other comparisons were not significant (*p* > 0.05).

In relation to last reported pornography use, when comparing the sexes, females reported higher scores than males in all mental health parameters. *Post hoc* analysis indicated significantly higher depression, anxiety and stress scores (all *p* < 0.001) in females than males who viewed pornography in the previous year. Additionally, females reporting having never used pornography also scored higher in anxiety levels than males in the same category (*p* < 0.05), while the scores for stress only showed a tendency toward significance (*p* = 0.06). Finally, there was a tendency toward significance (*p* = 0.07) in the difference between females and males in the anxiety scores for those reporting using pornography *More than a year ago*.

#### Mental Health and Recent Pornography Use

Given that the DASS-21 asks participants to consider the applicability of a specific statement over the **past week**, D, A, S scores were analyzed from participants responding that the last time they viewed pornography was *Within the past week* or *Today*, and that they viewed pornography at least weekly (*Multiple times daily*, *Daily*, or *Weekly*) (Used) relative to those who never used pornography (Not Used).

Analysis indicated a significant effect of pornography use in all three mental health parameters [D: *F*(1,527) = 45.98; A: *F*(1,527) = 21.08; S: *F*(1,527) = 21.96; all *p* < 0.001]. There was also a significant difference across sex for anxiety [*F*(1,527) = 5.37, *p* < 0.05] and stress [*F*(1,527) = 7.59, *p* < 0.01], but not depression [*F*(1,527) = 3.40, *p* > 0.05]. Additionally, none of the interactions pertaining to sex and pornography use were significant [D: *F*(1,527) = 0.23; A: *F*(1,527) = 0.38; S: *F*(1,527) = 0.13; all *p* > 0.05]. Depression, anxiety, and stress scores (mean and SEM), for both males and females, who used and did not use pornography are shown in the Figures 5D–F.

Within both males and females, there was a significant difference in depression (both *p* < 0.001), anxiety (Males: *p* < 0.001; Females: *p* < 0.01) and stress (Males: *p* < 0.01; Females: *p* < 0.001) scores between those who used (Used) and did not use (Not Used).

When comparing males and females, analysis revealed that within those who used pornography (Used), females reported significantly higher stress scores (*p* < 0.05) than males; however, there was no significant difference between the sexes in depression and anxiety scores (both *p* > 0.05) within Used. In participants who reported never using pornography, females scored significantly higher in anxiety (*p* < 0.05), but not depression (*p* > 0.05). There was also a tendency toward significance between males and females in stress scores within Not Used (*p* = 0.05).

### Multiple Regression Analysis

Regression analysis indicated various relationships between the various demographics (age, sex, number of semesters completed at Franciscan University, and whether or not the participant shared a room) analyzed, various aspects of pornography use (the last time the participant viewed pornography, the frequency of pornography use, the time of day at which they most often viewed pornography, and age of first exposure to pornography), including the aspects measured by the mCIUS and EmSS, and depression, anxiety and stress. Detailed effect sizes (β-values) with their *p*-values are shown in the Tables 4, 5.

**TABLE 4:** Influence of various demographics, including in relation to pornography use, and the modified Compulsive Internet Use Scale (mCIUS) variables on depression, anxiety and stress scores, measured using the DASS-21.

**Variable**		**Depression**			**Anxiety**			**Stress**		
		**β**	***P***	**Backward step of elimination**	**β**	***p***	**Backward step of elimination**	**β**	***P***	**Backward step of elimination**
Age		**−0.510**	**0.021**	**Not eliminated**	–0.355	0.071	16	–0.033	0.895	2
Sex		**−4.795**	**<0.001**	**Not eliminated**	**−3.755**	**<0.001**	**Not eliminated**	**−4.963**	**<0.001**	**Not eliminated**
Semesters		–0.294	0.443	6	–0.087	0.801	3	–0.070	0.838	4
Shareroom		–1.312	0.243	9	0.761	0.449	8	–0.199	0.841	3
Lastviewed		**1.802**	**<0.001**	**Not eliminated**	0.535	0.125	15	**1.267**	**<0.001**	**Not eliminated**
Frequent		–0.382	0.382	7	–0.222	0.574	6	–0.465	0.254	10
Timeday		–0.832	0.346	8	–0.251	0.753	4	–0.739	0.353	12
AgeFirstExp		–1.082	0.101	16	**−1.980**	**<0.001**	**Not eliminated**	**−1.377**	**0.026**	**Not eliminated**
Preoccupation	TimeOthers	0.291	0.677	3	–0.127	0.840	2	0.498	0.441	9
	ShortSleep	0.674	0.181	12	**1.249**	**0.002**	**Not eliminated**	0.588	0.242	11
	ThinkSites	0.379	0.555	5	0.462	0.409	9	0.906	0.114	13
	LookFwd	–0.806	0.101	15	–0.409	0.392	12	–0.580	0.242	14
	RushWork	–0.056	0.940	1	–0.109	0.869	1	0.289	0.669	7
	NglctOb	**1.240**	**0.009**	**Not eliminated**	0.327	0.523	7	**1.120**	**0.016**	**Not eliminated**
	Restless	0.580	0.264	11	**1.020**	**0.013**	**Not eliminated**	**1.559**	**<0.001**	**Not eliminated**
Dependence	DiffStop	–0.149	0.786	2	**−0.981**	**0.017**	**Not eliminated**	–0.773	0.084	15
	AccessStop	–0.639	0.250	10	0.395	0.463	11	–0.382	0.492	8
	SpendLess	–0.661	0.106	14	–0.423	0.249	14	–0.133	0.746	5
	Unsuccess	0.315	0.572	4	–0.433	0.387	10	–0.053	0.921	1
Emotional coping	FeelDown	1.136	0.142	13	0.522	0.224	13	0.663	0.118	16
	EscpSor	**1.747**	**<0.001**	**Not eliminated**	0.215	0.745	5	0.292	0.672	6
*R*^2^ for model		0.163			0.109			0.149		

*Effect sizes (β values) were obtained through backward stepwise regression analyses, as detailed in section “Materials and Methods.” Table shows the β value of each variable at the step in which it was eliminated from the model and the overall *R*^2^ for each model.*

**TABLE 5:** Influence of various demographics, including those associated with pornography use, and the emotional and sexual state variables on depression, anxiety and stress scores, measured using the DASS-21.

**Variable**		**Depression**			**Anxiety**			**Stress**		
		**β**	***P***	**Backward step of elimination**	**β**	***p***	**Backward step of elimination**	**β**	***P***	**Backward step of elimination**
Age		**−0.484**	**0.028**	**Not eliminated**	–0.342	0.085	13	–0.023	0.913	5
Sex		−**4.420**	**<0.001**	**Not eliminated**	−**4.068**	**<0.001**	**Not eliminated**	−**4.772**	**<0.001**	**Not eliminated**
Semesters		–0.180	0.638	6	–0.016	0.962	2	–0.007	0.984	2
Shareroom		–1.643	0.141	11	0.321	0.753	3	–0.048	0.964	3
Lastviewed		**1.866**	**<;0.001**	**Not eliminated**	0.510	0.148	12	**1.089**	**0.003**	**Not eliminated**
Frequent		–0.037	0.927	1	–0.128	0.716	6	–0.225	0.547	7
Timeday		–0.716	0.405	9	–0.006	0.994	1	–0.821	0.308	10
AgeFirstExp		–1.020	0.124	12	−**1.740**	**0.004**	**Not eliminated**	−**1.277**	**0.041**	**Not eliminated**
Interoceptive	Alone	–0.233	0.740	4	–0.205	0.746	4	0.321	0.627	6
	Lonely	**2.682**	**<0.001**	**Not eliminated**	**1.017**	**0.007**	**Not eliminated**	**1.905**	**<0.001**	**Not eliminated**
	Bored	–0.389	0.398	10	0.159	0.710	5	0.440	0.305	12
	Aroused	0.380	0.427	8	**0.858**	**0.035**	**Not eliminated**	**1.126**	**0.009**	**Not eliminated**
Impotent	Nosex	–0.057	0.896	2	0.225	0.561	7	0.003	0.995	1
	Noonesex	0.714	0.058	13	–0.381	0.285	10	–0.404	0.272	11
Extrinsic	Sexpart	0.196	0.665	5	–0.204	0.618	8	–0.367	0.386	8
	Peerpres	0.347	0.467	7	0.429	0.304	11	0.384	0.390	9
	Drunk	–0.146	0.737	3	0.382	0.309	9	0.028	0.946	4
*R*^2^ for model		0.157			0.091			0.144		

*Effect sizes (β values) were obtained through backward stepwise regression analyses, as detailed in section “Materials and Methods.” Table shows the β value of each variable at the step in which it was eliminated from the model and the overall *R*^2^ for each model.*

Both model 1 (that including mCIUS items) and model 2 (that including EmSS items) indicated that the participant’s age, sex, and the last time they viewed pornography significantly predicted depression scores (model 1: *R*^2^ = 0.163, model 2: *R*^2^ = 0.157). Sex and age of first exposure to pornography predicted both anxiety (model 1: *R*^2^ = 0.109, model 2: *R*^2^ = 0.091) and stress (model 1: *R*^2^ = 0.149, model 2: *R*^2^ = 0.144) scores. Additionally, the last time the participant viewed pornography was also a significant predictor for stress.

Pertaining to the specific items within the mCIUS (model 1), NglctOb and EscpSor significantly predicted depression scores, while DiffStop, ShortSleep, and Restless significantly predicted anxiety, and NglctOb and Restless were significant predictors of stress scores.

In relation to the EmSS items (model 2), Lonely significantly predicted all three mental health parameters measured (D, A, S). Additionally, Aroused was a significant predictor of both anxiety and stress, but not depression scores.

### Additional Information

#### What Helped Decrease Pornography Use

In relation to the question regarding what helped the participant decrease their pornography use, the “*Other (please specify)*” answer choice (*n* = 66) was excluded from analyses and percentages shown, due to the variety and ambiguity of responses given, which could potentially confound interpretation.

The overall distribution of responses in regards to aspects that helped decrease pornography use was as follows: *Internet resources (i.e. CovenantEyes.com)* (18.2%), *Accountability Partner/Group – on campus* (10.9%), *Accountability Partner/Group – off campus* (14.7%), *Faith life* (80.1%), *Moral principles* (76.6%), *Personal motivation* (81.2%), *Counseling services* (8.3%), *Nothing has helped* (3.9%), and *Not interested in decreasing use* (5.5%).

Both males and females reported *Faith life* (Males: 83.5%; Females: 76.2%), *Moral principles* (Males: 77.4%; Females: 75.7%), and *Personal motivation* (Males: 82.3%; Females: 79.9%) as the most helpful aspects in decreasing pornography use. These three options were not significantly different (all *p* > 0.05) from each other in both males [χ^2^(8, *N* = 243) = 1017.4, *p* < 0.001] and females [χ^2^(8, *N* = 214) = 1000.9, *p* < 0.001]. However, in both sexes, the proportions of participants reporting these options as sources of help were significantly higher than all other answer choices (all *p* < 0.001). Interestingly, 63.9% of respondents to this question included the combination of all three of these options (*Faith Life*, *Moral principles*, and *Personal motivation*) as sources that helped decrease pornography use.

In males, the proportions reporting internet resources (23.5%) and accountability partner both on- (16.5%) and off-campus (20.2%) were significantly higher than both those reporting that nothing had helped (4.9%) and those indicating that they were not interested in decreasing their use of pornography (4.1%; all *p* < 0.001). Additionally, the percentage of males reporting that counseling services (9.1%) helped in decreasing their use was significantly lower than both internet resources (*p* < 0.001) and accountability partner off-campus (*p* < 0.01). All other comparisons for males were not significant (all *p* > 0.05). Like males, a significantly higher proportion of females reported that internet resources (12.1%) helped reduce pornography use than those reporting that nothing helped decrease their pornography use (2.8%, *p* < 0.01). All other comparisons for females were not significant [*Accountability Partner/Group – on campus* (4.7%), *Accountability Partner/Group – off campus* (8.4%), *Counseling services* (7.5%), *Not interested in decreasing use* (7.0%); all *p* > 0.05].

There were no significant differences in the proportion of males and females reporting moral principles [χ^2^(1, *N* = 457) = 0.1, *p* > 0.05] and personal motivation [χ^2^(1, *N* = 457) = 0.3, *p* > 0.05] as sources of help in decreasing pornography use. However, there was a tendency toward significance in faith life [χ^2^(1, *N* = 457) = 3.4, *p* = 0.06]. The percentages of males reporting internet resources [χ^2^(1, *N* = 457) = 9.0, *p* < 0.01] and accountability partner on- and off-campus [χ^2^(1, *N* = 457) = 15.0 and 11.6, respectively, both *p* < 0.001] were significantly higher than females. All other comparisons were not significant [χ^2^(1, *N* = 457) = 0.2, 0.9, and 1.3 for *Counseling services*, *Nothing has helped*, and *Not interested in decreasing use*, respectively, all *p* > 0.05].

#### Perception of Struggle With Pornography on Campus

Regarding the questions inquiring into the percentage of male and female students the participants thought struggled with pornography on our campus, the most frequently selected choice was *50–74%* in relation to the percentage of males (41.4%) and *25–49%* in relation to the percentage of females (41.8%). 11.6, 31.4, and 15.7% of participants indicated that they thought *0–24*, *25–49*, and *75–100%* of males on campus struggled with pornography, respectively. Moreover, in relation to the percentage of females believed to struggle with pornography on our campus, *0–24%* was the second most selected answer choice (39.6%), followed by *50–74%* (16.9%) and *75–100%* (1.7%). A detailed breakdown of male and female perception of struggle with pornography on campus, in both sexes, is outlined in the Supplementary Table 3.

#### Perception of Level of Pornographic Content

Pertaining to the question regarding how pornographic the respondents considered various materials, *Moderately pornographic* (Mod) and *Extremely pornographic* (Ext) were the two highest answer choices for *Nude pictures (e.g., Playboy)* (Mod: 37.3%, Ext: 50.4%), *Erotic literature* (Mod: 44.0%, Ext: 31.3%), *Sexually explicit videos* (Mod: 10.6%, Ext: 86.8%), and *Cinematic sex scenes* (Mod: 40.2%, Ext: 37.4%). In regards to *Nude art (e.g., Statue of David, Sistine Chapel)*, *Not at all pornographic* (73.4%) and *Mildly pornographic* (21.4%) were the most selected answer choices. Additionally, 49.4 and 29.3% of participants reported *Seductive advertisements (e.g., Victoria’s Secret)* as *Mildly pornographic* and *Moderately pornographic*, respectively. The full details of how pornographic the participants considered the various materials to be, as well as sex differences in perception, are provided in the Supplementary Table 4.

## Discussion

The relationship between pornography use, compulsivity and mental health is complex and potentially multidirectional in terms of causality and the various subcomponents that constitute each individual variable. As indicated in the Introduction, one important variable is the increased use and accessibility of the internet for sexually related activities, which has become the major form of pornography consumed, most especially among younger individuals (Döring, 2009; Döring et al., 2017; Solano et al., 2020). Our study sought to investigate these variables in a sample of university students, in the hope of providing a better understanding of the dynamics of this relationship. In general, the results appear to indicate distinct and significant sex differences relative to both pornography use and the effect of such use on mental health. Moreover, the analysis also appears to highlight certain traits that seem to bear significant similarities to aspects of behavioral addictions, which also impact mental well-being.

As per previous reports (Carroll et al., 2008; Willoughby et al., 2014), our study appears to indicate a significant number of university students who reported lifetime pornography use. Significantly more males than females reported using pornography, more recently and more frequently, with the pre-teen (9–13) years being the primary period of first exposure to pornography in males. While this time-period of first exposure was also significant in females, in contrast to males, it extended into the adolescent (14–17) years. Another distinction between males and females is that while, in both cases, the majority of participants were exposed to pornography prior to the age of 18, the percentage of males in this category was significantly higher than that of females. While both sexes reported the same two primary methods of first exposure, they were distinct in that more females were exposed unintentionally, while more males were exposed through personal curiosity. Additionally, both sexes reported the cell phone as the primary method of access and adult websites as the primary form of pornography they were first exposed to and continued to access most often.

In relation to compulsive internet pornography use and emotional and sexual states associated with such use, the proportion of males was consistently higher in the items that displayed significant sex differences with the exception of the item pertaining to viewing pornography when with a sexual partner, where the proportion of females was higher. Our findings also appear to indicate that the items addressing compulsive pornography use and the emotional and sexual states involved in such use that were most predominantly reported by both sexes, pertained to components associated with dependence, emotional coping and interoception. However, the items pertaining to preoccupation and interoception were the items that most predicted mental health outcomes.

### Mental Health

Similar to our previous work (Beiter et al., 2015), a considerable number of students in this study reported symptoms indicative of severe and extremely severe depression, anxiety and stress, with percentages increasing from previous years. As evident in the scientific literature, efforts have never ceased to investigate potential contributors to the increasing number of reports of psychopathologies among university students, as well as potential ways to curtail the problem. The goal of our study was to contribute further to the body of literature by investigating the relationship of pornography use, as well as specific elements of the associated behavior in relation to compulsive use, and its potential to influence university student mental health.

Our results contribute to the current literature which indicates a potential link between pornography use and decreased mental well-being in female adolescents (Dalby et al., 2018), as well as lower psychosocial functioning in university students who reported higher levels of internet pornography addiction behaviors (Harper and Hodgins, 2016). Additionally, while previous research has also indicated a relationship between mental health and *perceived* addiction to pornography, as well as the influence of moral and religious/spiritual beliefs (Grubbs et al., 2015a,b,c, 2018, 2019; Bradley et al., 2016; Wilt et al., 2016), our study sought to establish a foundation for the investigation of the potential relationship between pornography use and addiction, through the measurement of actual behaviors reported to reflect compulsivity, which is a component of addiction (Meerkerk et al., 2009).

### Impaired Control

The original development of the CIUS (Meerkerk et al., 2009) was specifically based on addiction literature and the similarity that exists between compulsive internet use and addictive behaviors. While similar at various levels (Grant et al., 2006; Potenza, 2009; Kim and Hodgins, 2018), behavioral addictions differ from substance use addiction as they reflect pathological patterns of a specific behavior rather than the use of a specific substance to achieve the desired outcome/feeling (Grant et al., 2010; Potenza, 2014; Pinna et al., 2015). The adaptation of the original CIUS, by Downing et al. (2014), allowed for the use of the scale for assessment of compulsive use of internet pornography. While excessive pornography use is characterized under the category of behavioral addictions, but is not a diagnostic criterion within the Fifth Edition of the Diagnostic and Statistical Manual of Mental Disorders (DSM-V; American Psychiatric Association, 2013), various behaviors related to compulsive use of pornography are described by the International Classification of Disease manual (ICD-11; World Health Organization, 2018) classification for Compulsive Sexual Behavior Disorder.

#### Preoccupation

In substance use disorders, relating to compulsive use, preoccupation or the anticipation/craving of the substance is described under Criterion 4, in the section pertaining to Substance Use Disorders in the DSM-V (American Psychiatric Association, 2013), as well as in the scientific literature (Koob and Volkow, 2009). Our analyses appear to corroborate the presence of a factor that reflects the preoccupation aspect, represented by behaviors such as rushing work in order to access pornography websites, thinking about the websites when not online and the anticipation of the next internet pornography session.

#### Dependence

Additional aspects reflecting impaired control are the mCIUS items pertaining to finding it difficult to stop using pornography websites, continuing to access the websites despite the intention to stop, thinking that less time should be spent on the pornography websites and unsuccessfully trying to spend less time on the websites, which appear to reflect a level of dependence on or attachment to pornography. These behaviors are also reflective of those observed in substance use disorders (American Psychiatric Association, 2013), specifically, behaviors involving repeated efforts to minimize or discontinue use and an excessive time spent using.

#### Risky Behaviors

As previously mentioned, pornography use has also been associated with an increased involvement in high-risk sexual behaviors, including an association with an increased number of hook up partners, oral sex and sexual intercourse during a hookup, sexual permissiveness, anal intercourse, number of sexual partners, engaging in extramarital sex, and in paying for sex (Baggaley et al., 2010; Weinberg et al., 2010; Brody and Weiss, 2011; Morgan, 2011; Poulsen et al., 2013; Wright, 2013a,b; Braithwaite et al., 2015; Stannah et al., 2020). While it was beyond the scope of our study to directly address the prevalence of such risky behaviors in our sample, the aspects pertaining to the *Extrinsic* factor, including being more likely to view internet pornography when under the influence of alcohol or drugs and being with a sexual partner or peer-pressured appear to reflect circumstances with a potential to predispose the individual to vulnerable situations involving sexually risky behaviors (Lane et al., 2004; Camchong et al., 2014; Yang et al., 2019).

### Social Impairment and Isolation

The DSM-V (American Psychiatric Association, 2013) considers social impairment relative to substance abuse as consisting of a failure in the fulfillment of various essential life obligations (e.g., work, school, home), as well as a reduction in various important social, occupational or recreational activities. Our findings indicated similar behaviors among students reporting some level of lifetime pornography use, including preference toward accessing pornography over spending time with others, neglecting daily obligations due to preferring to access pornography, and rushing through work in order to access the websites. These behaviors, related to a preoccupation with pornography use, indicate a negative influence of such use on the individual’s normal daily functioning, including social behavior, revealing a similarity of compulsive use of internet pornography and behaviors associated with addiction.

Additionally, compulsive internet pornography use has also been shown to be associated with an increased level of isolation (Green et al., 2012). This is evident in the responses to the EmSS items inquiring about when the pornography was more likely to be viewed, specifically, the number of respondents who indicated that they were more likely to view pornography when alone or feeling lonely. The relationship between pornography and addiction, however, is complex. Butler et al. (2018) reports that the relationship between pornography consumption and loneliness is bidirectional. It is possible that relationship distress due to pornography use increases loneliness, while loneliness encourages pornography consumption due to its potential use as a coping mechanism. This is reflected in the findings of Popovic (2011) indicating that those who consume greater amounts of pornography demonstrate a higher craving for intimate relationships. Related to this are the EmSS items grouped under the factor labeled as *Impotent*, which reflect pornography use in situations associated with reduced possibilities of being able to engage in sexual intercourse.

Additionally, our analyses appear to highlight an emotional coping component of pornography use through the factor incorporating the mCIUS items relating to the access of pornography websites when feeling down or to escape/get relief from negative feelings. Moreover, the isolation experienced, resulting from pornography use, is not simply at the interoceptive level, but extends externally to also negatively influence relationships. As a result, it is unsurprising that pornography consumption is associated with loneliness (Yoder et al., 2005; Butler et al., 2018; Tian et al., 2018).

### Life Factors, Pornography Use, and Mental Health

The primary goal of this study was to address the relationship between pornography use and mental health, seeking to investigate whether compulsive pornography use is a potential contributor to the reduced mental well-being observed on university campuses. As previously mentioned, our results appear to corroborate previous literature indicating the presence/influence of sex differences in both mental health and various factors related to pornography consumption.

Early life factors impact the expression of and capacity to address depression, anxiety and stress. However, our results appear to indicate a distinction between depression, which was predicted by the current age of the participants, and anxiety and stress, which were predicted by age of first exposure to pornography, but not the current age of the participants. Relative to depression, it is possible that this may reflect research indicating the conglomeration of various factors that culminate in expression toward the end of the teen years, followed by a decline in subsequent years (Hankin, 2015; Kwong et al., 2019). It is possible that the distinction that exists in relation to anxiety and stress, being predicted by the age of first exposure to pornography, may be related to a certain specificity and longitudinal relationship to specific stressful events that are potentially indicative of an altered anxiety sensitivity. Anxiety sensitivity has been reported to be a significant mediator for the development of anxiety symptoms, but not depression (McLaughlin and Hatzenbuehler, 2009). A potentially similar mechanism may be taking place in regards to the relationship between age of first exposure and stress (Grasso et al., 2013; Tyborowska et al., 2018).

More directly related to pornography use, our study indicated that the last time pornography was viewed predicted both depression and stress, but not anxiety. Additionally, our results indicated that the primary items within the mCIUS that predicted all three mental health parameters (D, A, S) were related to some aspect of preoccupation with pornography use. Specifically, neglecting of obligations in order to view pornography significantly predicted both depression and stress, which appears to indicate the presence of significant distress or functional impairment, pertaining to the diagnosis of depression (American Psychiatric Association, 2013).

Additionally, similar to the clinical expression of anxiety (American Psychiatric Association, 2013), feelings of restlessness/frustration/irritation when unable to access pornography websites significantly predicted both anxiety and stress. Furthermore, an additional predictor of anxiety, associated with an aspect of preoccupation, was the shortness of sleep due to watching pornography, corroborating previous research relating insufficient sleep with increased expression of anxiety (Silva et al., 2004; Sagaspe et al., 2006; Ben Simon and Walker, 2019). In addition to the items that related to preoccupation, the use of pornography to alleviate negative feelings, bearing a similarity to reports of substance use to self-medicate in efforts to relieve negative affective symptoms (Bolton et al., 2009; Torres and Papini, 2016), also predicted depression scores. Moreover, similarities to substance use disorders also appear to be present in relation to finding it difficult to stop using pornography when online, potentially reflecting a level of dependence-related anxiety (Smith and Book, 2008).

Both items within the EmSS predicting mental health scores were related to the interoceptive factor. Specifically, viewing pornography when lonely predicted depression, anxiety and stress. Previous research has indicated that loneliness is associated with a physiological decline and involves interoceptive dysregulation (Arnold et al., 2019), which in turn, appears to be a significant component of various mental health conditions (for review see Khalsa et al., 2018). Additionally, the observed relationship between viewing pornography when feeling lonely and anxiety and depression may also be potentially mediated by some level of self-disgust (Ypsilanti et al., 2020), which may contribute to the expression of a desire to stop viewing pornography. An aspect of self-disgust may also be related to pornography’s relationship to a negative self-image (Stewart and Szymanski, 2012; Sun et al., 2014; Tylka, 2015), which in itself has been related to negative mental health outcomes (Gillen, 2015; Duchesne et al., 2016).

While interoceptive awareness has been positively correlated with sexual arousal (Nobre et al., 2004; Berenguer et al., 2019), the relationship between viewing pornography when feeling sexually aroused and negative mental health symptoms, such as anxiety and stress, appears to imply that, in pornography use, the arousal is potentially associated with an aspect of dysregulated interoception.

### Factors Assisting With Reducing Pornography Use

Given the previously reported negative effects of pornography, our study also sought to investigate potential resources that those using/having used pornography utilize/utilized and perceived to assist them in reducing pornography use.

Our results appear to suggest an influence of faith, morality and personal motivation on efforts to decrease pornography use. Previous research has indicated that factors such as self-motivation, mindfulness, religiosity and spirituality positively influence mental health (Yonker et al., 2012; Vitorino et al., 2018; Fountoulakis and Gonda, 2019; O’Driscoll et al., 2019). Additionally, higher levels of religiosity have been shown to be associated with a lower frequency of pornography consumption (Poulsen et al., 2013; Perry and Hayward, 2017). However, pertaining to the spiritual/religious aspect, previous work has also indicated the importance of a genuine application of the spiritual/religious life in order to avoid “spiritual bypass” (Welwood, 1984), which can be detrimental to recovery (Cashwell et al., 2007, 2009). Thus, given these observed relationships and the negative impact of pornography on the mental health parameters measured in our study, it appears that efforts directed at assisting persons affected by pornography should consider the potential incorporation of a genuine faith life and a moral foundation, as well as efforts to enhance traits associated with a healthy personal motivation in any treatment offered.

### Pornography Use and Coronavirus Disease 2019 (COVID-19)

While this study was conducted prior to the COVID-19 pandemic, it is important to also consider the relevance of our findings in relation to the reported increase in pornography use that took place from early March through mid-April 2020, with a worldwide peak increase of 24.4% being reported on March 25th (US peak: 41.5%; European peak: 18.0%) (Pornhub, 2020), as well as the efforts made to encourage engagement in sexual behaviors that minimize personal contact (Turban et al., 2020). This increase, potentially stress-related (e.g., as a result of isolation), is also particularly relevant in possibly contributing to negative coping mechanisms associated with problematic/pathological reinforcement patterns through the usage of internet-related technologies (Kiraly et al., 2020; Mestre-Bach et al., 2020). Pertaining specifically to university students, the potential impact of the lockdowns associated with the COVID-19 pandemic on the concepts investigated and discussed in our study are of very direct relevance, not only from the perspective of an increased potential pathological coping due to the increased stress associated with the changes necessary, but also from the perspective of the increased time spent on the computer and online, necessitated by the need to continue with classes.

## Limitations

As with all human studies, given the complexity of human behavior and the potential uniqueness of our sample, as well as the fact that our study involved participants from a single location, caution is necessary in relation to generalizability and various limitations exist that require consideration for both the interpretation of the results, as well as the direction of future studies. This, however, needs to be taken in the context of the consistency that exists between our results and those reported in both national and international studies. As with all surveys utilizing self-reporting, there is potential for recall bias. While efforts were made in some analyses to focus on specific very recent time points, the potential for recall bias cannot be dismissed and should also be taken into consideration in the interpretation of the results. Given that previous research (Fisher and Barak, 2001; Salmon and Diamond, 2012; Bishop, 2015; Chet et al., 2018; Lung et al., 2018) appears to indicate some distinction in the impact of various genres of pornography (e.g., violent vs. non-violent, paraphiliac vs. non-paraphiliac, heterosexual vs. homosexual theme etc.) on the user, among the limitations of this study is the fact that no distinction was made to separate the nature of the pornography used. While our study investigated the frequency of an individual’s pornography use, we did not address or distinguish between durations of the individual sessions (e.g., 1 h once a month vs. 5 h once a month). Additional aspects that were not addressed include (1) the potential financial burden associated with the use of pornography, (2) the potential role of the level of a person’s current faith and morals impacting an individual’s perception of pornography, and (3) specifics pertaining to behaviors associated with pornography use. In relation to the potential resources reported to have helped decrease pornography use, our results appear to highlight the necessity for a more detailed breakdown of specific factors within the specific resources listed in this study (e.g., Faith life: attending religious services, increased spiritual reading, etc.). Additional investigation is necessary to ensure a better understanding of the role of various resources, including faith, that can potentially assist in promoting positive mental health, through both quantitative and qualitative (including through the use of in-depth interviews) studies. Additionally, the results of this research indicate that future studies should potentially take into consideration the necessity of giving the opportunity to address, at the clinical level, any concerns pertaining to potential mental health consequences associated with pornography use.

## Conclusion

The necessity to understand the impact of pornography is broad due to its capacity to potentially influence various fundamental elements of society, including social interaction, human relationships and their integrity (e.g., fidelity, relationship satisfaction), human behavior (e.g., isolation, loneliness), and psychological well-being (e.g., partner distress) (e.g., Charny and Parnass, 1995; Bridges et al., 2003; Maddox et al., 2011; Minarcik et al., 2016).

Of concern is the potential capacity for pornography to influence sexual scripts through normalizing the observed behaviors (Tsitsika et al., 2009), which may potentially be related to an increased tolerance toward or acceptance of degrading/aggressive/violent sexual behaviors including, but not limited to, rape and sexual assault (Gerger et al., 2007), among both men (Foubert et al., 2011) and women (Norris et al., 2004).

In conclusion, our study highlights the interrelationship between pornography use and negative mental health outcomes in university/college students, predicted by compulsive behaviors reflecting behavioral addiction, indicating the potential for a relationship to the underlying neurobiological mechanisms present in addictive behaviors (Kuhn and Gallinat, 2014, 2015). Additionally, our findings provide some indication of potential resources that can be offered for consideration for reducing pornography use and the potential negative mental health consequences. Given the differences observed between the sexes, continued efforts are necessary to better understand the effects of pornography on the individual sexes, as well as to better understand potentially different effective treatments for each sex.

We believe that future studies should consider these findings, seeking to enhance the focus of attention and providing additional clarity on the impact of pornography on mental health and its similarity to addictive behaviors.

## Data Availability Statement

The datasets are available upon request. The raw data supporting the conclusions of this article will be made available by the authors, without undue reservation, to any qualified researcher.

## Ethics Statement

The studies involving human participants were reviewed and approved by the Franciscan University of Steubenville Institutional Review Board. The patients/participants provided their written informed consent to participate in this study.

## Author Contributions

SS supervised the study. SS and CC contributed to the conception, design of the study, and conducting of the study. SS, CC, and JP performed the statistical analyses. SS, CC, and JP wrote the first draft of the manuscript. All the authors contributed to manuscript revision, read and approved the submitted version.

## Conflict of Interest

The authors declare that the research was conducted in the absence of any commercial or financial relationships that could be construed as a potential conflict of interest.
