# Supplementary figures and images for: Compulsive Internet Pornography Use and Mental Health: A Cross-Sectional Study in a Sample of University Students in the United States
Source: Front Psychol. 2021 Jan 12;11:613244. doi: 10.3389/fpsyg.2020.613244 (PMC7835260; doi:10.3389/fpsyg.2020.613244)

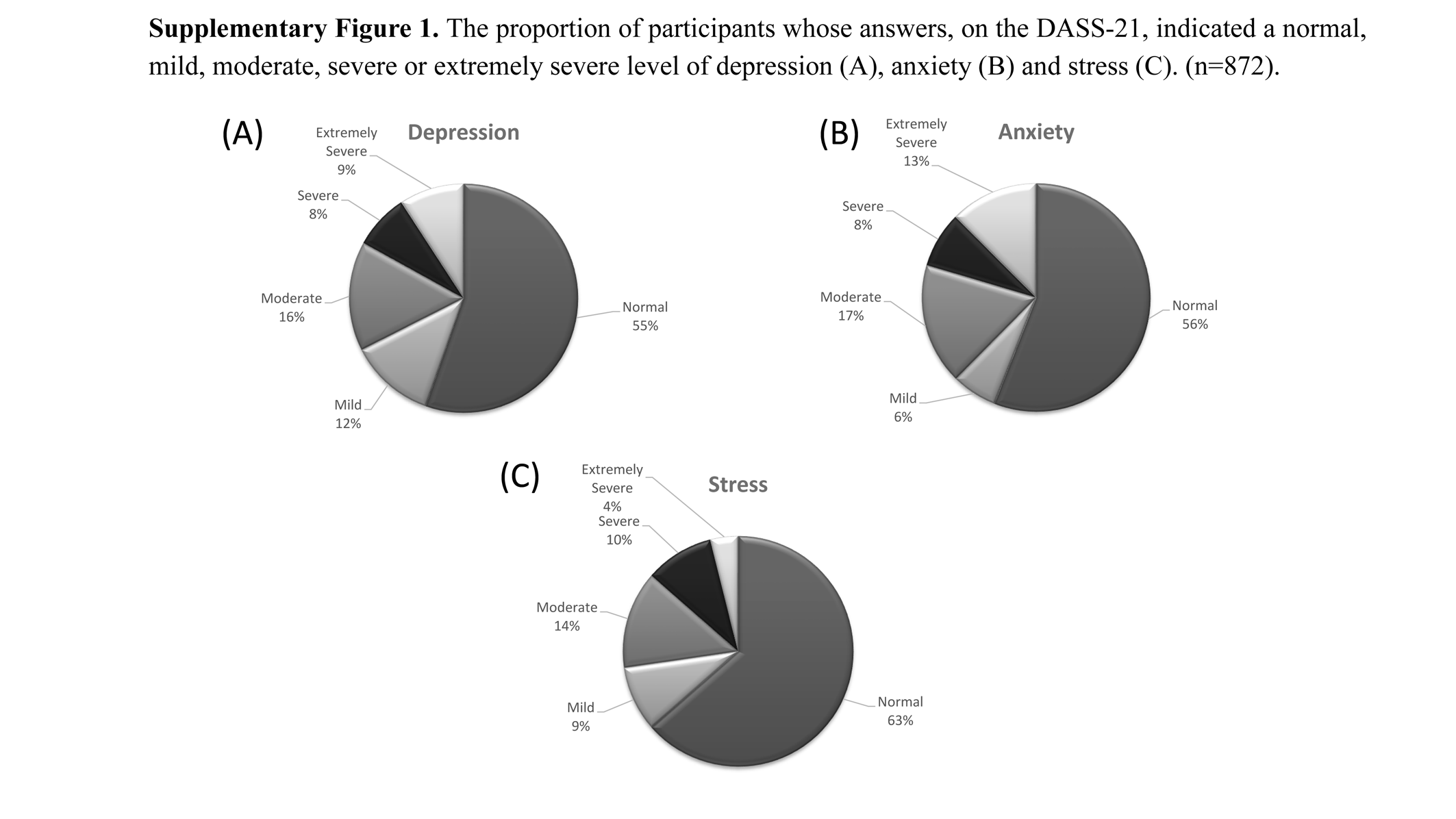

Supplement: Supplementary file 1 [file Image_1.TIF]
