# Supplementary material for: Compulsive Internet Pornography Use and Mental Health: A Cross-Sectional Study in a Sample of University Students in the United States
Source: Front Psychol. 2021 Jan 12;11:613244. doi: 10.3389/fpsyg.2020.613244 (PMC7835260; doi:10.3389/fpsyg.2020.613244)
Supplement: Supplementary file 2 [file Table_1.DOCX]

**Supplementary Table 1. Total percentage of students responding “Often” or “Very Often” to questions relating to compulsive pornography use across the sexes.** Items taken from modified Compulsive Internet Use Scale*; DiffStop*: Difficulty to stop accessing pornography websites, *AccessStop*: Access despite intention to stop, *TimeOthers*: Access pornography over spending time with others, *ShortSleep*: Short of sleep due to pornography use, *ThinkSites*: Think about websites when not online, *LookFwd*: Look forward to next session of use, *SpendLess*: Think it is necessary to spend less time, *Unsuccess*: Unsuccessful at spending less time, *RushWork*: Rush work to view pornography, *NglctOb*: Neglect obligations due to pornography, *FeelDown*: Use pornography when feeling down, *EscpSor*: Use pornography to escape negative feelings, *Restless*: Restless/frustrated/irritated when unable to view pornography. (Total: n=488; Male: n=254, Female: n=234).

| **mCIUS Item** | **Male** | **Female** |
| --- | --- | --- |
| Diffstop | 48.4 | 33.8 |
| Accessstop | 52.0 | 38.0 |
| Timeothers | 11.0 | 10.3 |
| Shortsleep | 21.7 | 17.5 |
| Thinksites | 19.7 | 17.9 |
| Lookfwd | 16.5 | 13.7 |
| Spendless | 77.6 | 62.8 |
| Unsuccess | 48.0 | 32.5 |
| Rushwork | 7.1 | 7.3 |
| NglctOb | 16.1 | 9.8 |
| Feeldown | 55.9 | 41.5 |
| EscpSor | 48.4 | 35.0 |
| Restless | 22.0 | 17.5 |
