# Supplementary material for: Compulsive Internet Pornography Use and Mental Health: A Cross-Sectional Study in a Sample of University Students in the United States
Source: Front Psychol. 2021 Jan 12;11:613244. doi: 10.3389/fpsyg.2020.613244 (PMC7835260; doi:10.3389/fpsyg.2020.613244)
Supplement: Supplementary file 3 [file Table_2.DOCX]

**Supplementary Table 2. Total percentage of students responding “Agree” or “Strongly Agree” to questions relating to emotional and sexual states pertaining to the use of pornography across the sexes.** Abbreviations pertaining to using internet pornography when *Alone*: by themselves, *Lonely*: feeling lonely, *Sexpart*: with a sexual partner, *Bored*: feeling bored, *Peerpres*: peer pressured, *Nosex*: not having had sex in a while, *Aroused*: feeling sexually aroused, *Drunk*: drunk or under effects of drugs, and *Noonesex*: unable to find someone to have sex with. (Total: n=476; Male: n=250, Female: n=226).

| **EmSS Item** | **Male** | **Female** |
| --- | --- | --- |
| Alone | 97.2 | 91.2 |
| Lonely | 75.2 | 66.8 |
| Sexpart | 6.8 | 14.6 |
| Bored | 80.0 | 66.4 |
| Peerpres | 5.2 | 9.3 |
| Nosex | 28.0 | 29.7 |
| Aroused | 83.2 | 78.3 |
| Drunk | 20.0 | 14.6 |
| Noonesex | 16.8 | 15.5 |
