# Supplementary material for: Compulsive Internet Pornography Use and Mental Health: A Cross-Sectional Study in a Sample of University Students in the United States
Source: Front Psychol. 2021 Jan 12;11:613244. doi: 10.3389/fpsyg.2020.613244 (PMC7835260; doi:10.3389/fpsyg.2020.613244)
Supplement: Supplementary file 4 [file Table_3.DOCX]

**Supplementary Table 3: Perception, by each sex, of percentage of students struggling with pornography on campus, within each sex.** (Total: n=899; Male: n=285, Female: n=614).

| **Overall Perception of Male & Female Struggle (%)** | | |
| --- | --- | --- |
|  | **Male** | **Female** |
| 0-24% | 11.6 | 39.6 |
| 25-49% | 31.4 | 41.8 |
| 50-74% | 41.4 | 16.9 |
| 75-100% | 15.7 | 1.7 |

| **Male Perception of Male & Female Struggle (%)** | | | |  |
| --- | --- | --- | --- | --- |
|  | **Male** | **Female** | | |
| 0-24% | 8.1 | 40.4 | | |
| 25-49% | 20.7 | 38.6 | | |
| 50-74% | 42.8 | 18.9 | | |
| 75-100% | 28.4 | 2.1 | | |
|  |  | |  |  |
|  |  | |  |  |
| **Female Perception of Male & Female Struggle (%)** | | | |  |
|  | **Male** | **Female** | | |
| 0-24% | 13.2 | 39.3 | | |
| 25-49% | 36.3 | 43.3 | | |
| 50-74% | 40.7 | 16.0 | | |
| 75-100% | 9.8 | 1.5 | | |
