# Supplementary material for: Compulsive Internet Pornography Use and Mental Health: A Cross-Sectional Study in a Sample of University Students in the United States
Source: Front Psychol. 2021 Jan 12;11:613244. doi: 10.3389/fpsyg.2020.613244 (PMC7835260; doi:10.3389/fpsyg.2020.613244)
Supplement: Supplementary file 5 [file Table_4.DOCX]

**Supplementary Table 4. Perception of level of pornography of various materials**. *Nudpic*: Nude pictures (e.g. Playboy), *EroLit*: Erotic literature, *ExpVid*: Sexually explicit videos, *Sexscenes*: Cinematic sex scenes, *NudeArt*: Nude art (e.g. Statue of David, Sistine Chapel) , *SedAds*: Seductive advertisements (e.g. Victoria’s Secret). (Total: n=899; Male: n=285, Female: n=614).

| **Overall Perception of Level of Pornography of Various Materials (%)** | | | | | | | | | |
| --- | --- | --- | --- | --- | --- | --- | --- | --- | --- |
|  | | Nudpic | EroLit | | ExpVid | Sexscenes | | NudeArt | SedAds |
| Not at all | | 0.6 | 3.1 | | 0.1 | 2.7 | | 73.4 | 12.5 |
| Mildly | | 11.8 | 21.6 | | 2.6 | 19.8 | | 21.4 | 49.4 |
| Moderately | | 37.3 | 44.0 | | 10.6 | 40.2 | | 3.1 | 29.3 |
| Extremely | | 50.4 | 31.3 | | 86.8 | 37.4 | | 2.1 | 8.9 |
|  |  | | |  | | |  |  |  |
|  |  | | |  | | |  |  |  |
| **Male Perception of Level of Pornography of Various Materials** | | | | | | | | | |
| **Male** | | Nudpic | EroLit | | ExpVid | Sexscenes | | NudeArt | SedAds |
| Not at all | | 0.7 | 3.9 | | 0.4 | 3.9 | | 75.1 | 9.8 |
| Mildly | | 11.6 | 25.6 | | 1.8 | 20.4 | | 20.4 | 52.6 |
| Moderately | | 42.5 | 44.2 | | 10.9 | 40.7 | | 2.5 | 30.2 |
| Extremely | | 45.3 | 26.3 | | 87.0 | 35.1 | | 2.1 | 7.4 |
|  | |  |  | |  |  | |  |  |
| **Female Perception of Level of Pornography of Various Materials** | | | | | | | | | |
| **Female** | | Nudpic | EroLit | | ExpVid | Sexscenes | | NudeArt | SedAds |
| Not at all | | 0.5 | 2.8 | | 0.0 | 2.1 | | 72.6 | 13.7 |
| Mildly | | 11.9 | 19.7 | | 2.9 | 19.5 | | 21.8 | 47.9 |
| Moderately | | 34.9 | 44.0 | | 10.4 | 39.9 | | 3.4 | 28.8 |
| Extremely | | 52.8 | 33.6 | | 86.6 | 38.4 | | 2.1 | 9.6 |
